# Disinfection of human musculoskeletal allografts in tissue banking: a systematic review

**DOI:** 10.1007/s10561-016-9584-3

**Published:** 2016-09-24

**Authors:** J. Mohr, M. Germain, M. Winters, S. Fraser, A. Duong, A. Garibaldi, N. Simunovic, D. Alsop, D. Dao, R. Bessemer, O. R. Ayeni, on behalf of the Bioburden Steering Committee and Musculoskeletal Tissue Working group, Amber Appleby, Scott Brubaker, Jeannie Callum, Graeme Dowling, Ted Eastlund, Margaret Fearon, Marc Germain, Cynthia Johnston, Ken Lotherington, Ken McTaggart, Jim Mohr, Jutta Preiksaitis, Michael Strong, Kimberly Young, Jie Zhao, Gary Rockl, Karl Shaver, Louis Thibault, Jacynthe Tremblay

**Affiliations:** 1 Canadian Blood Services, 270 John Savage Ave., Dartmouth, NS B3B 0H7 Canada; 2 Héma-Québec, 1070 Sciences-de-la-Vie Avenue, Quebec, QC G1V 5C3 Canada; 3 Nelson Laboratories, 6280 South Redwood Road, Salt Lake City, UT 84123-6600 USA; 4 Department of Surgery, McMaster University, 293 Wellington St. N, Suite 110, Hamilton, ON L8L 8E7 Canada; 5 McMaster University Medical Centre, 1200 Main St W, Room 4E15, Hamilton, ON L8N 3Z5 Canada

**Keywords:** Musculoskeletal allograft, Tissue donation, Tissue banking, Bioburden

## Abstract

Musculoskeletal allografts are typically disinfected using antibiotics, irradiation or chemical methods but protocols vary significantly between tissue banks. It is likely that different disinfection protocols will not have the same level of microorganism kill; they may also have varying effects on the structural integrity of the tissue, which could lead to significant differences in terms of clinical outcome in recipients. Ideally, a disinfection protocol should achieve the greatest bioburden reduction with the lowest possible impact on tissue integrity. A systematic review of three databases found 68 laboratory and clinical studies that analyzed the microbial bioburden or contamination rates of musculoskeletal allografts. The use of peracetic acid–ethanol or ionizing radiation was found to be most effective for disinfection of tissues. The use of irradiation is the most frequently published method for the terminal sterilization of musculoskeletal allografts; it is widely used and its efficacy is well documented in the literature. However, effective disinfection results were still observed using the BioCleanse™ Tissue Sterilization process, pulsatile lavage with antibiotics, ethylene oxide, and chlorhexidine. The variety of effective methods to reduce contamination rate or bioburden, in conjunction with limited high quality evidence provides little support for the recommendation of a single bioburden reduction method.

**Electronic supplementary material:**

The online version of this article (doi:10.1007/s10561-016-9584-3) contains supplementary material, which is available to authorized users.

## Introduction

Tissue banking is a process in which allografts are recovered from a donor and stored (banked) for future use. Prior to storage, the tissue banks process the tissues to remove microbial contaminants and ensure safety of the allografts for transplantation. The allograft is defined as sterile when processes assure no microorganisms are present to a particular level of assurance (Vangsness et al. 2006). Disinfection methods cannot guarantee absolute sterility, but can achieve a sterility assurance level (SAL). The SAL represents the probability that a sample will be contaminated following disinfection (Tenholder et al. 2003).

Health Canada states that the disinfection of allografts must be performed, but recommendations on specific methods or requirements are not published. Each tissue bank may employ its own method to disinfect tissue (Health Canada 2013). These methods are subdivided into ‘disinfection methods,’ and ‘terminal sterilization methods’ (Lambert et al. 2011). Disinfection methods include chemical and antibiotic treatments that target microorganisms, whereas terminal sterilization methods typically include irradiation, ethylene oxide, or heat treatments and eliminate all living microorganisms to a particular level of assurance following treatment (Lambert et al. 2011).

Hundreds of thousands of tissue transplants are performed globally each year. Musculoskeletal allografts (including bone, cartilage, tendons and ligaments typically used in orthopaedic procedures) are the focus of a number of research groups aimed to improve allograft patient outcomes (Saha and Roy 2013). Similar to other allografts, the risk of contamination with dangerous pathogens is high, and safe transplantation requires multiple processing considerations (including aseptic tissue recovery and aseptic tissue processing) in order to optimize allograft recipient outcome. Musculoskeletal allografts have the added benefit over cardiovascular allografts as most musculoskeletal allografts are amenable to terminal sterilization processes (Lambert et al. 2011). These sterilization methods typically remove all biological components, but are more likely to disrupt allograft structure and function. Bone allografts in particular are transplanted to provide structural integrity and growth factors to facilitate healing, and are less susceptible to adverse effects from terminal sterilization processes (Kamiński et al. 2009). Similarly, lower irradiation of tendon allografts may have no adverse effect on its mechanical properties (Reid et al. 2010).

The purpose of this systematic review is to determine the optimal methods of disinfection of musculoskeletal tissue following recovery to minimize the risk of disease transmission while maintaining the structure and function of the tissue for its intended use.

## Methods

### Information sources and search

The search strategy was developed and reviewed by McMaster and the Musculoskeletal Tissue Processing and Validation Subgroup (through SF). The electronic databases MEDLINE and EMBASE were searched from 1974 to May 29, 2014 using the following headings and text words: “musculoskeletal system,” “transplantation,” “bone transplantation,” “bone marrow transplantation,” “cartilage transplantation,” and “anti-fungal agent.” An additional reviewer (AH) performed a second search using the original search strategy in the Pubmed database to include publications up to March 6, 2015. The search included publications in English and excluded animal studies, case reports, conference abstracts and patent literature. The full search strategy is shown in Appendix A (Electronic Supplementary Material).

### Study selection

Seven reviewers (MW, SF, LT, MG, JT, GR, RP) independently screened each of the citations in duplicate to identify studies that met all of the following inclusion criteria: (1) evaluated musculoskeletal tissue (including bone, tendons, connective tissue, cartilage, and muscle), (2) evaluated any method during tissue processing to reduce bioburden, and (3) evaluated bioburden, tissue viability or transplantation results as outcomes. A study was excluded if it was an animal study, a case report or an editorial, letter, or review. If there was disagreement, the full report was retrieved and independent assessment was repeated until consensus was reached.

### Data abstraction

Design of data abstraction forms and evidence tables were guided by the questions in the analytic framework [Appendix B (Electronic Supplementary Material)] and approved and finalized by the musculoskeletal tissue subgroup (through SF). Four reviewers (AD, DD, RB and DA) independently abstracted the following study characteristics: first author, year of publication, country, sample size, donor, recovery site, tissues collected, pre-recovery sanitization (environment and donor), amount and type of recovered tissue, post-recovery storage conditions, and preservation methods. The microbial sample testing method was summarized for each study. Data collected for the outcomes included microbes detected immediately following tissue recovery, bioburden immediately following tissue recovery, antimicrobial intervention following initial bioburden assessment, incubation parameters, tissue integrity and proportion of allografts discarded or potentially discarded due to contamination as well as transplantation outcomes where applicable. All data abstraction was checked by the senior reviewers (AD and AG) to ensure accuracy and consistency.

### Quality assessment

Following the screening process, clinical studies that met the eligibility criteria were evaluated for quality using the Grades of Recommendation, Assessment, Development, and Evaluation (GRADE) assessment. The GRADE assessment analyzes a study’s limitations, inconsistency of results, indirectness of evidence, imprecision, and reporting bias and evaluates the quality of its evidence, thus allowing for informed recommendations (Guyatt et al. 2011). By systematically addressing multiple components that impact the quality of evidence, the GRADE approach facilitates criticism of the studies. There is no validated quality assessment tool for laboratory-based studies because basic science research is inherently considered level IV, or low quality evidence (Balshem et al. 2011).

### Data analysis

Data abstracted from all included studies were organized into tables presenting study characteristics, culture methods, and outcomes. Descriptive statistics included the frequency and percentage of bioburden outcomes, as well as mean proportions. A meta-analysis was not performed as there was high heterogeneity among the included clinical studies.

## Results

### Study selection

**Fig. 1:**
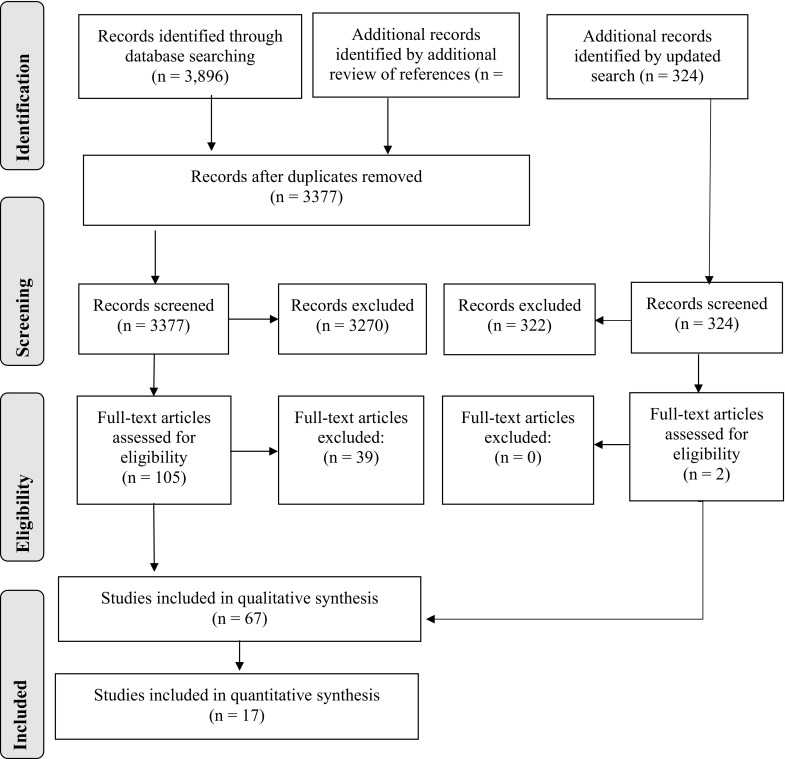
Summary of search strategy

A total of 3377 citations were reviewed after duplicates were removed (Fig. 1). Of the 3377 citations, 3270 were excluded because they did not fulfill the screening criteria. The full text articles of the 105 citations were retrieved for further evaluation. Sixty-six studies with a disinfection method and bioburden or tissue integrity as an outcome were included. The 39 studies that were excluded and the reasons for exclusion are provided in Appendix C (Electronic Supplementary Material). Following the updated search to include articles up to March 6, 2015, an additional 324 articles were retrieved and 2 were identified for further evaluation, and ultimately, inclusion. A final number of 68 articles, including 56 laboratory studies and 12 clinical studies were therefore included in this review.

### Quality of clinical studies

Of the 12 clinical reports, two studies performed prospective, randomized clinical trials, and are categorized as providing level I evidence (Sun et al. 2009, 2011). Two prospective cohort and one randomized trial were classified as level II evidence, whereas the remaining seven reports performed retrospective chart reviews or cohort studies (level III evidence) (Galia et al. 2009; Guo et al. 2012; Khoo et al. 2006; Krasny et al. 2013; Pruss et al. 2002). Using the GRADE assessment, the quality of clinical studies according to the objectives was found to range from very low to high. The clinical studies that addressed bioburden reduction loads were of very low quality. The clinical studies addressing terminal sterilization methods with irradiation had an average rating of low to moderate, whereas the one study which evaluated the BioCleanse™ Tissue Sterilization Process was found to have a high quality rating. The clinical studies that addressed the most effective parameters for disinfecting bone during tissue processing, as well as the patient outcomes related to use of irradiated bone, had an average rating of low to moderate for both of these findings. In relation to patient outcomes related to use of irradiated tendons, the relevant clinical studies were of moderate quality.

### Characteristics and culture methods of the studies

All included studies were conducted from 2000 to 2014, with the majority of laboratory studies conducted in Europe (26/56 studies), North America (26/56 studies), and the highest proportion of clinical studies conducted in Asia (5/12). A total of 4372 musculoskeletal samples were used in 56 laboratory studies, and 610 samples were used in 12 clinical studies. Thirty-one studies utilized cadaveric tissue and 8 studies used tissue recovered from organ donors. The type of donor was not indicated in the remaining 32 studies.

Recovery of tissues was performed in operating theatres for organ donors(Haimi et al. 2008; Schubert et al. 2012) and autopsy rooms for cadaveric donors (Lomas et al. 2004; Pruss et al. 2002), when reported. Two studies indicated recovery of cadaveric tissues in processing labs (Guo et al. 2012; Kaminski et al. 2012). Twelve studies indicated that during recovery, allografts were recovered under aseptic conditions, but did not address specific methods to reduce the bioburden. Only one study reported the use of class C and D air filters to reduce potential contamination of tissues by airborne contaminant (Kaminski et al. 2012).

The majority of studies analyzed bone tissues exclusively (35/68), whereas 14.7 % (10/68) of the studies analyzed tendon, ligament and cartilage tissue. Of these studies, researchers analyzed both bone and tendons in 23 studies (32.4 %). Following recovery of the tissue, saline and Hank’s Balanced salt solution were the only reported solutions for short term storage. Seven studies chose to preserve their tissue samples at temperatures lower than −40 °C.

Tissue samples may become contaminated before, during or following recovery, which poses a threat to the allograft recipient. Researchers used a variety of techniques to determine the bioburden, or the amount of contaminating microorganisms (bacteria, fungi, or viruses). Culturing of sample swabs or of the sample directly was used to detect bacteria and fungi in 15 laboratory studies, and two clinical studies. Growth media for detection of bacteria and fungi were extremely diverse and included Muller-Hinton agar, soybean casein agar (also called tryptic soy agar), tryptic soy broth, horse blood agar, Schaedler’s broth, and Kanamycin-Esculin agar. The same media (blood agar media), was used for both the detection of fungi and bacteria in two studies. The detection of viruses was performed by viral cell culture in 10 studies (infection, and subsequent growth of infected cells containing viruses). A microbiological assay to detect the bioburden was not reported for 34 laboratory studies, and 10 clinical studies. Incubation of bacterial cultures between 30 and 37 °C for 1–10 days were reported in 16 studies.

### Study outcomes

#### Bioburden and microbe identification

Prior to intervention, the contamination rate ranged from 0 to 10.1 % in three studies that reported the initial bioburden (Dunsmuir and Gallacher 2003; Parker and Maschke 2008; Schubert et al. 2012). A method to describe the cleaning of the musculoskeletal allograft was reported in 32 laboratory studies, and six clinical studies. Of the reported methods, mechanical cleaning to remove additional tissue, and irrigation were the most common methods of cleaning following recovery. In six studies, chemical agents such as dimethylformamide with Fmoc-[2-(2amino-ethoxy)-ethoxy]-acetic acid, hydrogen peroxide, and chloroform–methanol solutions were used to remove additional soft tissue, such as fat or other debris from the allografts. The contamination rate following tissue cleaning was only reported in one study.

In Schubert et al. (2012), the presence of a number of contaminating bacteria and fungi were observed. These included species, in descending order of prevalence, such as coagulase negative *Staphylococcus*, *Micrococcus*, *Bacillus cereus*, *Corynebacterium*, *Penicilium*, *Alcaligenes*, *Lactobacillus*, *Eschericcia coli*, *Acinetobacter*, *Enterococcus*, *Pseudomonas aeruginosa*, *Pneumococcus*, and the fungus, *Candida.* These authors noted that of these species, *Streptococcus*, *Acinetobacter*, *and Escherichia* are highly virulent (Schubert et al. 2012). A number of studies performed tests on tissues that were contaminated by researchers following recovery to assess the efficacy of their decontamination methods. Similar to the recovered contaminating microorganisms observed in Schubert et al. (2012), the most commonly inoculated organisms in these studies were *Staphylococci* and *Bacillus* species. Seven studies sought to test various decontamination methods on tissues infected with viruses, such as HIV2, pseudorabies virus (PRV), bovine virus diarrhoea virus (BVD), hepatitis A virus (HAV), and porcine parvovirus (PPV).

#### Bioburden reduction

For the laboratory studies, two studies were able to eliminate all viruses and bacteria using peracetic acid and ethanol soak for 2–4 h, and were effective in reducing the logarithmic bioburden >4.19 to >8.23-fold. This treatment revealed the greatest reduction in bioburden compared to all other decontamination methods. Parker and Maschke (2008) demonstrated that pulsatile lavage of the allografts with a solution containing polymyxin B (166.66 units/cc) and bacitracin (16.66 units/cc) reduced the logarithmic bioburden by an average of 2.1 fold, whereas the mechanical agitation or soaking in the same antibiotic solution was only capable of reducing the logarithmic bioburden by an average of >1.5 and 0.7-fold, respectively.

In the clinical studies, the bioburden was only addressed in one report. In Pruss et al. (2002), treatment of allografts with peracetic acid–ethanol reduced the contamination rate to 0 %. The GRADE quality of evidence for both studies were determined to be very low. The remainder of the clinical studies did not report the bioburden or contamination rate following treatment.

Irradiation, peracetic acid, and BioCleanse™ Tissue Sterilization Processes were used to treat the allografts in the clinical studies.

In contrast to the disinfection methods, the majority of papers utilized terminal sterilization methods to reduce the bioburden. The greatest reduction in the contamination rate was observed when samples were irradiated at 25 kGy, or treated with ethylene oxide or heat (82.5 °C). When exposed to 25 kGy of irradiation, three studies found that all bacteria and viruses were eliminated (Baker et al. 2005; Hilmy et al. 2007; Nguyen et al. 2011). At 30 kGy, the logarithmic viral (HIV) bioburden reduction was 4.2 fold, but treatment of samples with 50 kGy of irradiation followed by an additional 30 kGy treatment resulted in the greatest logarithmic reduction in bioburden (>8.2 fold log reduction) (Hernigou et al. 2000). Irradiation at 800 W for more than 2 min also reduced the viral contamination rate to 0 % (Dunsmuir and Gallacher 2003).

One study was also able to eliminate all viruses from contaminated bone samples using an ethylene oxide treatment, reducing the logarithmic bioburden by >5.3 fold (Moore et al. 2004). Heating of samples at 82.5 °C reduce the logarithmic viral bioburden by > 4.26 fold (Pruss et al. 2003b).

#### Tissue structural integrity

Irradiation was performed in 16 laboratory studies. In the majority of the studies, treatment of the allografts with less than 25 kGy did not affect the integrity of the allograft. Irradiation temperature was reported in only three studies, including −50, 30, 50, and 80 °C (Grieb et al. 2005; Hernigou et al. 2000; Pruss et al. 2002). Researchers assessed a number of different variables, including cyclic elongation and stiffness of the tendons, or strength and elastic modulus, and failure load acceptance of the bone allografts. At or above 25 kGy, the mechanical effects are varied, but in general, there is a dose-dependent effect, with increasing irradiation dosages. Seven studies showed that there was a reduction in tensile strength (acoustic emission), maximum force, and deformation energy with exposure to large doses of irradiation (>35 kGy). Interestingly, two studies showed an increase in ultimate strain, Young’s modulus, and strain energy density or the resilience and elastic limit of bones, following irradiation at 18–50 kGy, and 35 kGy, respectively (Grieb et al. 2005; Kaminski et al. 2012).

Tissue viability following treatment with ethylene oxide was addressed in one report. Lomas et al. (2001) reported that this treatment with aeration eliminated all viruses, but induced production of the inflammation cytokines, TNF-α and IL-6. To reduce cytokine induction, the researchers disinfected the allograft with ethylene oxide without aeration, and were still able to achieve full disinfection of the allograft (Lomas et al. 2001). Treatment without aeration is more likely to cause physiological issues due to the residual ethylene oxide (Lomas et al. 2001). Tissue viability was not assessed directly following the use of other chemicals to disinfect the allografts.

#### Transplantation outcomes

Prior to release for transplantation, a portion of allografts from the donor are tested to ensure that they are free of bacteria. Of the clinical studies, eight reports utilized a total of 606 irradiated allografts for transplantation, and most studies disinfected the samples using 25, 35, or 50 kGy of irradiation. Of these, eight transplanted allografts were found to be infected following transplantation (1.3 %). The source of the infection was not stated. One was resolved by treatment with antibiotics, and another required amputation (Khoo et al. 2006). The GRADE quality of the clinical evidence was low to moderate.

Of the remaining studies, 43 BioCleanse™ Tissue Sterilization Processed allografts, 154 antibiotic-treated allografts, and 3087 peracetic acid–ethanol treated allografts were transplanted into patients (Indelicato et al. 2013; Pruss et al. 2002). No recipients experienced any primary infections as a result of contaminated allografts from the BioCleanse™ or peracetic acidethanol-treated allografts; however, nine peracetic acid–ethanol treated transplant recipients did experience secondary infections (Indelicato et al. 2013; Pruss et al. 2002). Approximately 3.9 % of patients (6/154) receiving the antibiotic-treated allografts developed primary deep wound infections. The source of the infections were not stated. The GRADE quality of the BioCleanse™ Sterilization Process studies was high.

In addition to primary outcomes regarding infection rate in transplant recipients, three studies also addressed secondary outcomes as a result of irradiation at 25 kGy (Guo et al. 2012; Sun et al. 2009, 2011). Performing comparison of irradiated to non-irradiated allografts, recipients with irradiated allografts report more anterior laxity, and significant differences in the Lachman test, ADT, pivot shift test, and instrumented KT-2000 arthrometer tests. The GRADE quality of evidence, regarding the tendon and bone integrity was rated as low to moderate for the studies in bone, and moderate for the tendon analysis. The remainder of the studies reported no significant differences in secondary outcomes or did not report secondary outcomes at all.

### Confounding effects

The use of antibiotics, irradiation, and chemical sterilization have greatly improved the number of recovered musculoskeletal allografts available to patients. However, it is difficult to assess the efficacy of the majority of these methods, without baseline indicators, such as the initial bioburden or contamination rate following recovery. A limited number of reports have addressed this by quantifying bioburden, and representing the logarithmic reduction in bioburden in the results. Similarly, a number of studies report methods to clean the allografts following recovery. Without measurements prior to, and immediately following these methods, it is difficult to assess the best practices to clean allografts following recovery.

An additional confounding effect is the diverse number of parameters for the reported decontamination methods. For example, the majority of reports utilized irradiation at doses between 0.05 and 630 kGy. This extensive range allows for direct comparisons between irradiation dosages, but this was rarely addressed for other processing methods, such as the effect of temperature on disinfection efficiency.

## Discussion

The incidence of musculoskeletal allograft contamination can be as high as 10.1 %, and the studies in this review demonstrated a number of methods to address this issue. It was found that incubation of tissues in peracetic acid–ethanol for 4 h resulted in the greatest reduction in bioburden. Irradiation with 25 kGy was the most common method of terminal sterilization, and was effective in bioburden reduction, with minimal negative impact on tissue structural integrity. Most studies reported the long term preservation of allografts below −40 °C, and transplantation of irradiated, peracetic acid and ethanol-treated, or BioCleanse™ Tissue Sterilization Processed allografts resulted in no adverse events related to contamination.

Following recovery, the allografts often need to be cleaned to remove extraneous tissues. A number of studies reported cleaning processes to remove additional tissues, but bioburden was not assessed following cleaning.

All studies employed a method to disinfect the allografts. The most effective method to disinfect the tissue was chemical sterilization with peracetic acid–ethanol treatment. Following a 4 h incubation, observers reported the presence of almost no viral or bacterial contaminants, reducing the contamination rate to 0 %. One study reported that peracetic acid ethanol treatment had no effect on the presence of hepatitis A virus, suggesting that this species, or possibly even all hepatoviruses may be resistant to peracetic acid–ethanol treatment (Pruss et al. 2003a).

Other methods, such as antibiotic treatment or BioCleanse™ Tissue Sterilization Processing of the allografts were able to reduce bioburden, but were not as effective as percetic acid–ethanol treatment, when reported. It should be noted that while the use of antibiotics to soak the allografts was relatively effective in bioburden reduction, the use of pulsatile lavage using the same antibiotic-containing solution exponentially increased the reduction in bioburden. The details regarding the flow rate and method of pulsatile lavage were not reported, and its effectiveness in reducing bioburden may be increased with optimization of the protocol.

A number of reports opted to use terminal sterilization methods to reduce the bioburden. The most commonly reported method was irradiation. Both gamma irradiation and electron beam irradiation were used, and showed similar capacities in both bioburden reduction and maintenance of tissue following treatment. The greatest logarithmic reduction in bioburden (>8.2 fold) was observed when samples were exposed to 50 kGy of irradiation (Grieb et al. 2005). Although the studies addressed a wide range of radiation dosages, the majority of studies with effective reduction of bioburden with minimal effect on the allograft viability utilized a dosage ranging from 18 to 35 kGy.

Heat treatments were relatively ineffective at decontamination relative to irradiation and ethylene oxide treatment. Ethylene oxide (214 mg/dl) exposure at 25 °C for 4 h was able to completely eliminate the contamination rate to 0 % (Moore et al. 2004). Although tissue viability was not monitored at 25 °C, increasing the treatment temperature to 37 °C was also effective in decontamination and did not induce cytokine induction when aeration was excluded from the protocol (Lomas et al. 2001).

Terminal sterilization is not typically used for non-musculoskeletal allografts, as they may affect tissue integrity. Three studies revealed that irradiation results in significant differences in multiple tests to assay transplant function in the recipient. These include the Lachman test, ADT, pivot shift test, and instrumented KT-2000 arthrometer test (Guo et al. 2012; Sun et al. 2009, 2012). Peracetic acid–ethanol was used in one study to disinfect allografts, and posttransplantation assays revealed good clinical outcomes for all recipients (Pruss et al. 2002).

### Limitations

In this systematic review, the most effective methods of musculoskeletal allograft decontamination were assessed. As previously stated, 56 studies were laboratory studies and are not amenable to GRADE analysis. However, 12 clinical studies were included, and were evaluated for their level of evidence. Seven studies provided level III evidence, which are not typically used to formulate clinical recommendations. As clinical reports are usually formulated based on level I and II evidence.

The contamination rate and logarithmic bioburden reduction outcomes were utilized to determine the most effective methods of decontamination. There was a large amount of heterogeneity in the culturing methods to determine both of these values. Some organisms are extremely fastidious, and may only grow within a narrow range of nutrient and environmental conditions. Most studies used media types that are proposed to be able to capture the majority of organisms that may contaminate the tissues, but the use of only one culturing media, or incubation parameter could possibly exclude important pathogens that would affect transplantation outcomes. There was also a lack of data regarding clinical outcomes which were sought to address in this review.

The initial bioburden was reported in only one study, and as such, it is difficult to assess the best pre-recovery precautionary measures, or allograft cleaning methods to reduce bioburden. Additionally, the number of decontamination methods discussed was extremely diverse, but the reports failed to address a number of parameters that could affect positive outcomes following decontamination. One exception was the use of irradiation, which was comprehensively examined among the reports. Testing of irradiation to disinfect tissues featured a broad range of radiation dosages, as well as incubation conditions that allow for informed conclusions.

Antibiotics were used to disinfect allografts in three studies (Parker and Maschke 2008; Saegeman et al. 2009; Schubert et al. 2012). Although a number of different antibiotics were used, incubation conditions were similar within studies. This allows for direct comparisons of other factors, but may not necessarily represent optimal conditions for disinfection. Parker and Maschke (2008) was the only study that reported quantitative bioburden levels following treatment, as well as addressing different methods of administering antibiotics to allografts.

A similar trend was observed for chemical disinfection of the allografts. Chlorhexidine was used to disinfect tissues at concentrations ranging from 0.2 to 10 %. At higher concentrations, there was a corresponding increase in the number of samples that were free from contamination. However, there were no assays of tissue integrity, viability, or transplantation outcomes that suggest its safety or efficacy in preserving tissue integrity (Hernigou et al. 2000; Saegeman et al. 2009; Schubert et al. 2012). When samples were treated with ethylene oxide, there was a much narrower range of different conditions tested, but tissue integrity was assessed, which could also provide insight into protocol optimization (Bienek et al. 2007; Lomas et al. 2001; Moore et al. 2004).

Five laboratory studies and one clinical study opted to test the efficacy of the BioCleanse™ Tissue Sterilization Process. The use of this system was extremely effective in reducing the bioburden, and did not have any reported adverse effects on the tissue integrity or transplantation outcomes. The product and process is patented, and therefore does not allow for programs to adopt without licensing the technology.

Finally, a large proportion of the studies did not address the bioburden reduction capabilities of the disinfection methods used. In 13 laboratory studies and one clinical study, the reduction in bioburden as a result of the disinfection process was reported. As opposed to the contamination rate, the reduction in bioburden value can quantitatively show the effectiveness of disinfection methods, and allows for further optimization.

## Conclusions

The results of this review suggest that the use of peracetic acid–ethanol for 4 h, or terminal sterilization methods such as irradiation (<25 kGy), results in the greatest quantitative reduction in bioburden, with minimal effects on tissue viability and transplantation outcomes, BioCleanse™ Tissue Sterilization Processing demonstrated significant qualitative reduction in bioburden with minimal effects on tissue viability and transplantation outcomes. Long term storage of musculoskeletal allografts is often through freezing at temperatures below −40 °C. A limited number of reports suggest that pulsatile lavage with antibiotic solutions, or treatment with ethylene oxide or chlorhexidine may also be an effective method of bioburden reduction following optimization of these processes

## Electronic supplementary material

Below is the link to the electronic supplementary material.

Supplementary material 1 (PDF 128 kb)

Supplementary material 2 (PDF 45 kb)

Supplementary material 3 (PDF 90 kb)

Supplementary material 4 (PDF 120 kb)

Supplementary material 5 (PDF 105 kb)

Supplementary material 6 (PDF 369 kb)

Supplementary material 7 (PDF 92 kb)
