# Supplementary material for: Disinfection of human musculoskeletal allografts in tissue banking: a systematic review
Source: Cell Tissue Bank. 2016 Sep 24;17(4):573–84. doi: 10.1007/s10561-016-9584-3 (PMC5116033; doi:10.1007/s10561-016-9584-3)
Supplement: Supplementary file 1 — Supplementary material 1 (PDF 128 kb) [file 10561_2016_9584_MOESM1_ESM.pdf]

APPENDIX A: SEARCH STRATEGY

MEDLINE

1	exp Musculoskeletal System/tr [transplantaion]
2	Bone Transplantation/
3	exp "Bone and Bones"/tr [transplantation]
4	exp Connective Tissue/tr [Transplantation]
5	exp Cartilage/tr [Transplantation]
6	exp Muscles/tr [Transplantation]
7	(bone adj transplant*).mp.
8	(bone? adj2 (graft* or allograft* or allo-graft* or autogeneicgraft* or autogeneic-graft* or syngraft* or syn-graft* or syngeneicgraft* or syngeneic-graft* or homograft* or homo-graft*)).mp.
9	(connective tissue? adj3 transplant*).mp.
10	(connective tissue? adj3 (graft* or allograft* or allo-graft* or autogeneicgraft* or autogeneic-graft* or syngraft* or syn-graft* or syngeneicgraft* or syngeneic-graft* or homograft* or homo-graft*)).mp.
11	(cartilage* adj3 transplant*).mp.
12	(cartilage* adj3 (graft* or allograft* or allo-graft* or autogeneicgraft* or autogeneic-graft* or syngraft* or syn-graft* or syngeneicgraft* or syngeneic-graft* or homograft* or homo-graft*)).mp.
13	(musc?l* adj3 transplant*).mp.
14	(musc?l* adj3 (graft* or allograft* or allo-graft* or autogeneicgraft* or autogeneic-graft* or syngraft* or syn-graft* or syngeneicgraft* or syngeneic-graft* or homograft* or homo-graft*)).mp.
15	((osteoarticular or osteo-articular) adj3 transplant*).mp.
16	((osteoarticular or osteo-articular) adj3 (graft* or allograft* or allo-graft* or autogeneicgraft* or autogeneic-graft* or syngraft* or syn-graft* or syngeneicgraft* or syngeneic-graft* or homograft* or homo-graft*)).mp.
17	(musculoskeletal adj2 tissue?).mp.
18	or/1-17
19	Tissue Preservation/
20	Organ Preservation/
21	"Tissue and Organ Procurement"/
22	exp Anti-Bacterial Agents/
23	exp Antifungal Agents/
24	exp Cryoprotective Agents/
25	exp Sterilization/
26	Tissue Banks/
27	Bone Banks/
28	((tissue? or organ?) adj2 preserv*).mp.
29	((tissue? and organ?) adj2 procurement*).mp.
30	anti-bacterial agent?.mp.
31	antibacterial agent?.mp.
32	bacteriocid*.mp.
33	antifung* agent?.mp.

34 anti-fung* agent?.mp.
35 fungicid*.mp.
36 (cryoprotective adj2 agent?).mp.
37 (steriliz* or sterilis*).mp.
38 ((tissue? or bone?) adj2 (bank or banking)).mp.
39 (bioburden or bio-burden).mp.
40 or/19-39
41 18 and 40
42 limit 41 to (case reports or congresses or consensus development conference or consensus development conference, nih or duplicate publication)
43 41 not 42
44 limit 43 to yr="1970 -Current"
45 limit 44 to (english or french)

EMBASE

1 exp musculoskeletal system/
2 exp transplantation/
3 1 and 2
4 musculoskeletal procedure/
5 exp bone transplantation/
6 exp bone marrow transplantation/
7 muscle transplantation/
8 muscle graft/
9 exp cartilage transplantation/
10 (bone adj2 transplant*).mp.
11 (bone? adj5 (graft* or isograft* or iso-graft* or allograft* or allo-graft* or autograft* or auto-graft* or autogeneicgraft* or autogeneic-graft* or syngraft* or syn-graft* or syngeneicgraft* or syngeneic-graft* or homograft* or homo-graft*)).mp.
12 (musc?l* adj5 transplant*).mp.
13 (musc?l* adj5 (graft* or isograft* or iso-graft* or allograft* or allo-graft* or autograft* or auto-graft* or autogeneicgraft* or autogeneic-graft* or syngraft* or syn-graft* or syngeneicgraft* or syngeneic-graft* or homograft* or homo-graft*)).mp.
14 (cartilage* adj5 transplant*).mp.
15 (cartilage* adj5 (graft* or isograft* or iso-graft* or allograft* or allo-graft* or autograft* or auto-graft* or autogeneicgraft* or autogeneic-graft* or syngraft* or syn-graft* or syngeneicgraft* or syngeneic-graft* or homograft* or homo-graft*)).mp.
16 (connective tissue? adj5 transplant*).mp.
17 (connective tissue? adj5 (graft* or isograft* or iso-graft* or allograft* or allo-graft* or autograft* or auto-graft* or autogeneicgraft* or autogeneic-graft* or syngraft* or syn-graft* or syngeneicgraft* or syngeneic-graft* or homograft* or homo-graft*)).mp.
18 ((osteoarticular or osteo-articular) adj5 transplant*).mp.

19	((osteoarticular or osteo-articular) adj5 (graft* or isograft* or iso-graft* or allograft* or allo-graft* or autograft* or auto-graft* or autogeneicgraft* or autogeneic-graft* or syngraft* or syn-graft* or syngeneicgraft* or syngeneic-graft* or homograft* or homo-graft*)).mp.
20	(musculoskeletal adj2 tissue?).mp.
21	or/3-20
22	tissue preservation/
23	organ preservation/
24	cryopreservation/
25	exp antifungal agent/
26	cryoprotective agent/
27	((tissue? or organ?) adj2 preserv*).mp.
28	((tissue? and organ?) adj2 procurement*).mp.
29	(cryopreserv* or cryo-preserv*).mp.
30	anti-bacterial agent?.mp.
31	antibacterial agent?.mp.
32	bacteriocid*.mp.
33	antifung* agent?.mp.
34	anti-fung* agent?.mp.
35	fungicid*.mp.
36	(cryoprotective adj2 agent?).mp.
37	(steriliz* or sterilis*).mp.
38	((tissue? or bone?) adj2 (bank or banking)).mp.
39	(bioburden or bio-burden).mp.
40	or/22-39
41	21 and 40
42	(exp animals/ or exp animal experimentation/) not ((exp animals/ or exp animal experimentation/) and exp human/)
43	41 not 42
44	limit 43 to embase
