# Supplementary material for: Disinfection of human musculoskeletal allografts in tissue banking: a systematic review
Source: Cell Tissue Bank. 2016 Sep 24;17(4):573–84. doi: 10.1007/s10561-016-9584-3 (PMC5116033; doi:10.1007/s10561-016-9584-3)
Supplement: Supplementary file 2 — Supplementary material 2 (PDF 45 kb) [file 10561_2016_9584_MOESM2_ESM.pdf]

APPENDIX B: ANALYTICAL FRAMEWORK

Cleaning and Disinfection of Frozen Human Musculoskeletal Allografts
(i.e. bone, soft tissue, connective tissue, cartilage and osteoarticular tissue)

1. Does the method for manual cleaning of tissues impact bioburden load?
    a. What method(s) for cleaning and removing extraneous tissue, cells and lipids from musculoskeletal tissue results in the lowest bioburden load?

2. Do different decontamination methods result in different bioburden reduction loads (both qualitatively and quantitatively)?
    a. What decontamination method is most effective in reducing the bioburden content (load) during tissue processing? ***Effective*** *is defined as producing the highest reduction in bioburden both quantitatively and qualitatively.* Variables to compare:
        i. Mechanical cleaning of tissue
        ii. Chemical treatments
        iii. Rinse agents
        iv. Antibiotics
        v. Sonification
        vi. Centrifugation
        vii. Pressure cycles
    b. What are the most effective parameters for decontaminating bone during tissue processing in reducing bacterial content (load)? ***Effective*** *is defined as producing the highest reduction in bioburden both quantitatively and qualitatively.* Variables to compare:
        i. Concentrations
        ii. Combinations
        iii. Incubation temperature
        iv. Incubation duration
        v. Exposure times
        vi. Sequence of application

Final Sterilization of Musculoskeletal Tissue

3. Do different terminal sterilization methods result in different sterility assurance levels?
    a. What are the most effective methods for final (terminal) sterilization? ***Effective*** *is defined as achieving sterilization (a 6 log reduction in bioburden).* Variables to compare:
        i. Dry heat
        ii. Electron beam radiation
        iii. Gamma radiation
        iv. Ethylene oxide gas
        v. Moist heat
        vi. Supercritical CO_2

    b. What are the most effective parameters for final (terminal) sterilization to achieve a 6 log reduction? ***Effective*** *is defined as achieving sterilization (a 6 log reduction in bioburden).* Variables to compare:
        i. Radiation dose
        ii. Chemical concentration
        iii. Exposure time

4. Does the method of terminal sterilization impact the functionality of bone tissue?
    a. What are patient outcomes related to irradiated bone?

5. Does method of terminal sterilization impact the functionality of tendons?
    a. What are patient outcomes related to irradiated tendons?

Storage and Transportation

6. Do temporary storage parameters effect bioburden load?
    a. What are the most effective storage parameters for preventing and inhibiting microbial growth? ***Effective*** *is defined preventing proliferation of microbes.* Variables to compare:
        i. Storage method
        ii. Storage temperature
        iii. Transportation duration
