# Supplementary material for: Disinfection of human musculoskeletal allografts in tissue banking: a systematic review
Source: Cell Tissue Bank. 2016 Sep 24;17(4):573–84. doi: 10.1007/s10561-016-9584-3 (PMC5116033; doi:10.1007/s10561-016-9584-3)
Supplement: Supplementary file 3 — Supplementary material 3 (PDF 90 kb) [file 10561_2016_9584_MOESM3_ESM.pdf]

APPENDIX C:

Table 1: Excluded Studies

Excluded articles	Reason
Taylor, 2014	Commentary paper
Ang, 2014	Did not assess bioburden. Assumes sterility by analyzing core temperature of bone during sterilization
Atique, 2014	Tissue isolated from living donor
Burton, 2014	Tissue isolated from animal
Putzer, 2014	Tissue isolated from living donor
Reich, 2014	Tissue isolated from animal
Kaya, 2013	Tissue isolated from living donor
Nguyen, 2013	Tissue isolated from living donor
Singh 2012	Tissue isolated from living donor
Coraca-Huber, 2013	Aim was to look at chemical treatments for impregnation of antibiotics; did not assess bioburden; used *Bacillus subtilis* bioassay to assess gentamicin release only. Sterility is presumed from penetration (but not tested)
Moore, 2012	Tissue isolated from living donor
Villalba, 2012	Tissue isolated from living donor
Samsell, 2012	Review paper
Folsch 2012	Review paper
Schwiedrzik, 2011	Tissue isolated from living donor
Bauer, 2011	Tissue isolated from living donor
Ko, 2011	Study analyzed adipose tissue and mesenchymal stem cell allografts
Nyland, 2009	Tissue isolated from animal
Love, 2009	Tissue isolated from living donor
Kayurapan, 2009	Tissue isolated from animal
Costain 2009	Review paper
Thalgott, 2009	Patient outcomes of allografts not related to bioburden
Borkhuu, 2008	Antibiotic impregnated bones are out of scope.
Mahirogullari 2007	Review paper
Gajiwala, 2007	Case report
Laitinen, 2006	Tissue isolated from living donor
Brosig, 2005	Tissue isolated from living donor
Day, 2005	Lab study on antibiotic measurements in bone
Buttaro, 2005	Assessed antibiotic concentration on femoral heads in allograft recipients
Hirn, 2004	Tissue isolated from living donor
Pruss,Seibold et al 2003	Tissue isolated from living donor
Weyts, 2003	Isolation of human cells from allografts
Soyer, 2002	Tissue isolated from living donor
Parthiban, 2002	X-ray review examining auto- vs allograft disc space settling.
Kaku, 2002	Lab study examining methods to reduce ethylene oxide concentrations in bone following sterilization. Ethylene oxide may be harmful, and might affect transplantation outcome, but not studied.

Excluded articles	Reason
Hirn, 2001	Technical note on cleaning of morselized allograft.
Smith, 2001	No discussion of musculoskeletal tissue
Matter, 2001	Tissue isolated from animal donor
Molina, 2000	Tissue isolated from living donor
Sommerville, 2000	Tissue isolated from living donor
Winkler, 2000	Timecourse of vancomycin and tobramycin elution from loaded bone samples.

References of excluded articles:

Ang, C. Y., Yew, A., Tay, D., Chia, S. L., Yeo, S. J., Lo, N. N., & Chin, P. L. (2014). Reducing allograft contamination and disease transmission: intraosseous temperatures of femoral head allografts during autoclaving.*Singapore medical journal*, *55*(10), 526-528.

Atique, B., & MM, R. K. (2013). The bacterial contamination of allogeneic bone and emergence of multidrug-resistant bacteria in tissue bank. *BioMed research international*, *2014*, 430581-430581.

Bauer, J., Liu, R. W., Kean, T. J., Dennis, J. E., Petersilge, W., & Gilmore, A. (2011). A comparison of five treatment protocols for contaminated bone grafts in reference to sterility and cell viability. *The Journal of Bone & Joint Surgery*, *93*(5), 439-444.

Borkhuu, B., Borowski, A., Shah, S. A., Littleton, A. G., Dabney, K. W., & Miller, F. (2008). Antibiotic-loaded allograft decreases the rate of acute deep wound infection after spinal fusion in cerebral palsy. *Spine*, *33*(21), 2300-2304.

Brosig, H., Jacker, H. J., Borchert, H. H., Kalus, U., Dörner, T., von Versen, R., & Pruss, A. (2005). Sufficient penetration of peracetic acid into drilled human femoral heads. *Cell and tissue banking*, *6*(3), 231-237.

Burton, B., Gaspar, A., Josey, D., Tupy, J., Grynpas, M. D., & Willett, T. L. (2014). Bone embrittlement and collagen modifications due to high-dose gamma-irradiation sterilization. *Bone*, *61*, 71-81.

Buttaro, M. A., Pusso, R., & Piccaluga, F. (2005). Vancomycin-supplemented impacted bone allografts in infected hip arthroplasty TWO-STAGE REVISION RESULTS. Journal of Bone & Joint Surgery, British Volume, 87(3), 314-319.

Coraça-Huber, D. C., Hausdorfer, J., Fille, M., & Nogler, M. (2013). Effect of storage temperature on gentamicin release from antibiotic-coated bone chips. *Cell and tissue banking*, *14*(3), 395-400.

Costain, D. J., & Crawford, R. W. (2009). Fresh-frozen vs. irradiated allograft bone in orthopaedic reconstructive surgery. Injury, 40(12), 1260-1264.

Day, R. E., Megson, S., & Wood, D. (2005). Iontophoresis as a means of delivering antibiotics into allograft bone. Journal of Bone & Joint Surgery, British Volume, 87(11), 1568-1574.

Fölsch, C., Mittelmeier, W., Bilderbeek, U., Timmesfeld, N., von Garrel, T., & Matter, H. P. (2012). Effect of storage temperature on allograft bone.Transfusion Medicine and Hemotherapy, 39(1), 36.

Gajiwala, A. L., Kumar, B. D., & Chokhani, P. (2007). Evaluation of demineralised, freeze-dried, irradiated bone allografts in the treatment of osseous defects in the oral cavity. *Cell and tissue banking*, *8*(1), 23-30.

Hirn, M., Laitinen, M., Pirkkalainen, S., & Vuento, R. (2004). Cefuroxime, rifampicin and pulse lavage in decontamination of allograft bone. *Journal of Hospital Infection*, *56*(3), 198-201.

Hirn, M. Y., Salmela, P. M., & Vuento, R. E. (2001). High-pressure saline washing of allografts reduces bacterial contamination. *Acta Orthopaedica*,*72*(1), 83-85.

Kaku, N., Tsumura, H., Kataoka, M., Taira, H., & Torisu, T. (2002). Influence of aeration, storage, and rinsing conditions on residual ethylene oxide in freeze-dried bone allograft. *Journal of orthopaedic science*, *7*(2), 238-242.

Kaya, I., Sungur, I., Yilmaz, M., Pehlivanoglu, F., Kartyasar, K., & Sengoz, G. (2013). Comparison of the efficiency of different antibiotic irrigation solutions in decontamination of allografts contaminated with Staphylococcus aureus.*Acta orthopaedica et traumatologica turcica*, *47*(4), 281-285.

Kayurapan, A., Aresanasuwan, T., & Waikakul, S. (2009). Decreasing strength of bone allograft after recovery and preservation. *Journal of the Medical Association of Thailand= Chotmaihet thangphaet*, *92*, S76-80.

Ko, M. S., Jung, J. Y., Shin, I. S., Choi, E. W., Kim, J. H., Kang, S. K., & Ra, J. C. (2011). Effects of expanded human adipose tissue-derived mesenchymal stem cells on the viability of cryopreserved fat grafts in the nude mouse.International journal of medical sciences, 8(3), 231.

Love, D., Pritchard, M., Burgess, T., Van Der Meer, G., Page, R., & Williams, S. (2009). Audit of the Douglas Hocking Research Institute bone bank: ten years of non-irradiated bone graft. *ANZ journal of surgery*, *79*(1-2), 55-61.

Laitinen, M., Kivikari, R., & Hirn, M. (2006). Lipid oxidation may reduce the quality of a fresh-frozen bone allograft. Is the approved storage temperature too high?. *Acta orthopaedica*, *77*(3), 418-421.

Mahirogullari, M., Ferguson, C. M., Whitlock, P. W., Stabile, K. J., & Poehling, G. G. (2007). Freeze-dried allografts for anterior cruciate ligament reconstruction. Clinics in sports medicine, 26(4), 625-637.

Matter, H. P., Garrel, T. V., Bilderbeek, U., & Mittelmeier, W. (2001). Biomechanical examinations of cancellous bone concerning the influence of duration and temperature of cryopreservation. *Journal of biomedical materials research*, *55*(1), 40-44.

Molina, M. E., Nonweiller, D. E., Evans, J. A., & DeLee, J. C. (2000). Contaminated anterior cruciate ligament grafts: the efficacy of 3 sterilization agents. *Arthroscopy: The Journal of Arthroscopic & Related Surgery*, *16*(4), 373-378.

Moore, M. A. (2012). Inactivation of enveloped and non-enveloped viruses on seeded human tissues by gamma irradiation. *Cell and tissue banking*,*13*(3), 401-407.

Nguyen, H., Cassady, A. I., Bennett, M. B., Gineyts, E., Wu, A., Morgan, D. A., & Forwood, M. R. (2013). Reducing the radiation sterilization dose improves mechanical and biological quality while retaining sterility assurance levels of bone allografts. *Bone*, *57*(1), 194-200.

Nyland, J., Larsen, N., Burden, R., Chang, H., & Caborn, D. N. M. (2009). Biomechanical and tissue handling property comparison of decellularized and cryopreserved tibialis anterior tendons following extreme incubation and rehydration. *Knee Surgery, Sports Traumatology, Arthroscopy*, *17*(1), 83-91.

Parthiban, J. K., Singhania, B. K., & Ramani, P. S. (2002). A radiological evaluation of allografts (ethylene oxide sterilized cadaver bone) and autografts in anterior cervical fusion. *Neurology India*, *50*(1), 17.

Pruss, A., Seibold, M., Benedix, F., Frommelt, L., von Garrel, T., Gürtler, L., ... & Göbel, U. B. (2003). Validation of the ‘Marburg bone bank system’for thermodisinfection of allogenic femoral head transplants using selected bacteria, fungi, and spores. *Biologicals*, *31*(4), 287-294.

Putzer, D., Huber, D. C., Wurm, A., Schmoelz, W., & Nogler, M. (2014). The Mechanical Stability of Allografts After a Cleaning Process: Comparison of Two Preparation Modes. *The Journal of arthroplasty*, *29*(8), 1642-1646.

Reich, M. S., Kishore, V., Iglesias, R., & Akkus, O. (2014). Genipin as a sporicidal agent for the treatment of cortical bone allografts. *Journal of biomaterials applications*, *28*(9), 1336-1342.

Samsell, B. J., & Moore, M. A. (2012). Use of controlled low dose gamma irradiation to sterilize allograft tendons for ACL reconstruction: biomechanical and clinical perspective. Cell and tissue banking, 13(2), 217-223.

Schwiedrzik, J. J., Kaudela, K. H., Burner, U., & Zysset, P. K. (2011). Fabric-mechanical property relationships of trabecular bone allografts are altered by supercritical CO 2 treatment and gamma sterilization. *Bone*, *48*(6), 1370-1377.

Singh, R., & Singh, D. (2012). Sterilization of bone allografts by microwave and gamma radiation. *International journal of radiation biology*, *88*(9), 661-666.

Smith, R. A., Ingels, J., Lochemes, J. J., Dutkowsky, J. P., & Pifer, L. L. (2001). Gamma irradiation of HIV-1. *Journal of Orthopaedic Research*, *19*(5), 815-819.

Sommerville, S. M. M., Johnson, N., Bryce, S. L., Journeaux, S. F., & Morgan, D. A. F. (2000). Contamination of banked femoral head allograft: incidence, bacteriology and donor follow up. *Australian and New Zealand Journal of Surgery*, *70*(7), 480-484.

Soyer, J., Rouil, M., & Castel, O. (2002). The effect of 10% povidone–iodine solution on contaminated bone allografts. *Journal of Hospital Infection,50*(3), 183-187.
Taylor, S. A., & Marx, R. (2014). Cautious Optimism. The Journal of Bone & Joint Surgery, 96(16), e142.

Villalba, R., Peña, J., Navarro, P., Luque, E., Jimena, I., Romero, A., & Villagrán, J. G. (2012). Cryopreservation increases apoptosis in human menisci. *Knee Surgery, Sports Traumatology, Arthroscopy*, *20*(2), 298-303.

Weyts, F., Bos, P. K., Dinjens, W., van Doorn, W. J., van Biezen, F., Weinans, H., & Verhaar, J. (2003). Living cells in 1 of 2 frozen femoral heads. Acta Orthopaedica, 74(6), 661-664.

Winkler, H., Janata, O., Berger, C., Wein, W., & Georgopoulos, A. (2000). In vitro release of vancomycin and tobramycin from impregnated human and bovine bone grafts. *Journal of Antimicrobial Chemotherapy*, *46*(3), 423-428.
