# Supplementary material for: Disinfection of human musculoskeletal allografts in tissue banking: a systematic review
Source: Cell Tissue Bank. 2016 Sep 24;17(4):573–84. doi: 10.1007/s10561-016-9584-3 (PMC5116033; doi:10.1007/s10561-016-9584-3)
Supplement: Supplementary file 4 — Supplementary material 4 (PDF 120 kb) [file 10561_2016_9584_MOESM4_ESM.pdf]

Appendix D

Table 2: Laboratory Study Characteristics

First author, Year	Country	Number of Sites	Donor	Recovery Site	Pre-recovery sanitization (environment and donor)	Type of Recovered Tissue	Post Recovery Storage Conditions (short term)	Preservation Method
Elenes, 2014	USA	1	NR	NR	"Aseptically recovered"	Tendons- bone-patellar tendon-bone and tibialis tendons	NR	-70°C (fresh frozen)
Kaminski, 2012	Poland	1	Cadaveric	Processing lab	Environment-Class D and C air	Bone (femur)	-70°C	NR
Schubert, 2012	Belgium	1	Organ	Operating theaters or specific-tissue recovery room	NR	Bone and tendon	4°C	NR
Hernandez, 2012	United States	NR	Cadaveric	NR	NR	Bone (femur)	Packaged in dry ice	NR
Schmidt, 2012	Germany	1	NR	NR	NR	"principles of a tendon model"	NR	–78 °C
Shaw, 2012	USA	NR	Cadaveric	NR	NR	Bone- fibulae	Frozen	1 (control), 2, 4 or 8 freeze-thaw cycles (down to at least -40°C and up to 1 to 2°C)
Jung, 2011	USA	NR	Cadaveric	NR	NR	Patella, patellar tendon and at least 10 cm of tibial bone	Frozen (once only)	1 (control), 4 or 8 freeze-thaw cycles (down to -20°C (>6 h) and up to

First author, Year	Country	Number of Sites	Donor	Recovery Site	Pre-recovery sanitization (environment and donor)	Type of Recovered Tissue	Post Recovery Storage Conditions (short term)	Preservation Method
								22°C (>6 H)
Cornu, 2011	Belgium	NR	Cadaveric	NR	NR	Bone (femur)	NR	Freeze dried
Hoburg 2011	Germany	1	NR	NR	Accredited authorized sterilization site	Bone-patellar tendon-bone	Packaged in gas impermeable sterilization bags;-70°C.	-70°C
Barth, 2011	United States	1	Cadaveric	NR	NR	Bone (femur)	Refrigerated in Hanks' Balanced Salt Solution	NR
Nguyen, 2011	Australia	1	Living and cadaveric	Living-operating theatre; cadaveric-NR	Followed standard operating procedures	Bone (femoral heads; structure and morselized allografts)	NR	NR
Reid, 2010	United States	NR	Cadaveric	NR	NR	Bone-patellar tendon-bone	NR	Stored in freezer
Ketonis, 2010	United States	1	NR	NR	NR	Bone (cortical stubs or morselized bone)	NR	NR
Hoburg, 2010	Germany	1	NR	NR	NR	Bone-patellar tendon-bone	NR	Packaged in CO_2-filled gas-impermeable bags and frozen at -70C
Bitar, 2010	Brazil	1	Cadaveric	NR	NR	Semi-tendonous muscle tendons	0.9% NaCl	-4°C for up to 48 h, or -80°C for 40 d
Kaminski, 2009	Poland	1	Cadaveric	NR	According to tissue bank	Bone-patellar tendon-bone	NR	All allografts were stored at –70°C,

First author, Year	Country	Number of Sites	Donor	Recovery Site	Pre-recovery sanitization (environment and donor)	Type of Recovered Tissue	Post Recovery Storage Conditions (short term)	Preservation Method
					standard procedure			except for lyophilized tendons
Vastel, 2009	France	NR	Organ	NR	NR	Bone (femoral heads)	Saline	-40C
Saegeman, 2009	Belgium	NR	Organ and cadaveric	NR	NR	Bone	NR	NR
Balsly, 2008	United States	1	NR	NR	NR	Bone (cloward dowels and iliac crest wedges) and soft tissue (patellar tendons, anterior tibialis tendons, semitendinosus tendons, and fascia lata)	NR	-80°C; except fascia lata which were freeze-dried and packaged
Mikhael, 2008	United States	NR	Cadaveric	NR	NR	Bone (femur or tibia)	NR	-80C
Parker, 2008	United States	1	NR	Operating theatre	"Under sterile conditions per tissue bank protocol"	Bone-patellar tendon-bone	NR	–80°C
Kattaya, 2008	United States	1	NR	NR	NR	Bone (femur)	NR	NR
Nguyen, 2008	Australia	1	Living and cadaveric	Living-operating theatre; cadaveric-	Followed standard operating procedures	Bone (femoral heads, structural bone and milled bone allografts)	NR	NR

First author, Year	Country	Number of Sites	Donor	Recovery Site	Pre-recovery sanitization (environment and donor)	Type of Recovered Tissue	Post Recovery Storage Conditions (short term)	Preservation Method
				NR				
Haimi, 2008	Finland	1	Organ	Operating theatre	NR	Bone (tibia and femur)	NR	–75°C
Han, 2008	United States	2	Cadaveric	NR	NR	Bone	NR	NR
Giannini, 2008	Italy	NR	NR	NR	NR	Posterior tibial tendons	Fresh samples were processed or frozen	-80°C
Henson, 2008	USA	NR	NR	NR	NR	Tibialis posterior tendon	NR	-80°C after 2 h or 8 h in cryoprotectant
Lewis, 2008	USA	1	NR	NR	NR	Menisci	NR	Fresh frozen -80°C
Scheffler, 2007	Germany	1	NR	NR	"Recovered under aseptic conditions"	Soft tissue (Achilles tendon, skin and cartilage)	Frozen in liquid nitrogen	Freeze-dried
Vastel, 2007	France	NR	Cadaveric organ	NR	NR	Bone (femoral head)	NR	Saline at -40°C
Hilmy, 2007	Indonesia	Several hospitals	Cadaveric	NR	NR	Femoral head and ring, tibia ring, humerus, patella tendon, tendon, fascia lata, ribs, struct, amnion	NR	Freeze dried
Jones, 2007	USA	NR	Cadaveric	NR	NR	Bone-patellar tendon-bone allografts	NR	Temporary storage at -80°C and -20°C
Schimizzi, 2007	USA	NR	Cadaveric	NR	NR	Bilateral tibialis anterior tendons	On ice	-60°C

First author, Year	Country	Number of Sites	Donor	Recovery Site	Pre-recovery sanitization (environment and donor)	Type of Recovered Tissue	Post Recovery Storage Conditions (short term)	Preservation Method
Bienek, 2007	United Kingdom	NR	Cadaveric tissue donor	NR	NR	Bone- Femoral shafts	NR	NR
Grieb, 2006	USA	1	NR	NR	NR	Semitendinosus tendons	NR	-80°C
Mroz, 2006	USA	NR	Cadaveric	NR	NR	Bone- Cylindrical cortical pins from femur and tibia	NR	NR
Akkus, 2005 (1)	USA	NR	Cadaveric	NR	NR	Bone- Femurs	Saline + calcium	-40°C
Akkus, 2005 (2)	USA	NR	Cadaveric	NR	NR	Bone- Femoral shafts	Saline + calcium	-40°C
Scheffler, 2005	Germany	NR	Organ	NR	NR	Bone-patellar tendon-bone	-18°C	-35°C
Grieb, 2005	USA	1	NR	NR	NR	Bone- Cancellous bone dowels	NR	NR
Baker, 2005	USA	Multiple	NR	NR	NR	NR	NR	NR
Mitchell, 2004	USA	2	NR	NR	NR	Bone- Femurs	NR	NR
Lomas, 2004	United Kingdom	NR	Cadaveric tissue donor	Autopsy Room	"Aseptic techniques"	Patellar tendons	< -40°C	< -40°C
Moore, 2004	USA	NR	NR	NR	NR	Bone powder, femoral cortex, iliac wedge, cancellous cubes, patellar bone-tendon-bone ligament	NR	NR

First author, Year	Country	Number of Sites	Donor	Recovery Site	Pre-recovery sanitization (environment and donor)	Type of Recovered Tissue	Post Recovery Storage Conditions (short term)	Preservation Method
Vastel, 2004	France	NR	Cadaveric	NR	NR	Bone- Femoral heads	Frozen	-80°C in saline
Pruss,Kao et al 2003	Germany	1	NR	NR	NR	Bone- Femoral heads	NR	-70°C
Dunsmuir, 2003	United Kingdom	1	NR	NR	NR	Bone- Femoral heads	-80°C	-30°C
Pruss,Gobel et al 2003	Germany	NR	NR	NR	NR	Spongiosa tissue from vertebral column, and epiphyses of femurs or tibiae	NR	NR
Tenholder, 2003	USA	1	Cadaveric	NR	Routine, aseptic	Bone - femur	NR	NR
Dufrane, 2002	Belgium	NR	NR	Operating room or autopsy room	Instruments and equipment were sterilized	Fascia lata	Sterile physiological saline	Room temperature
Pruss, Kao, *et al*, 2002	Netherlands	1	Cadaveric	NR	NR	Cortical bone	NR	NR
Pruss, 2001	Germany	NR	NR	NR	"Sterile conditions"	Spongiosa tissue from columna vertebralis, and epiphyses of femur, humerus or tibia	NR	NR
Akkus, 2001	USA	1	NR	NR	NR	Bone- Cortical bone from femora	NR	Frozen

First author, Year	Country	Number of Sites	Donor	Recovery Site	Pre-recovery sanitization (environment and donor)	Type of Recovered Tissue	Post Recovery Storage Conditions (short term)	Preservation Method
Lomas, 2001	United Kingdom	1	Cadaveric	NR	NR	Bone- Femoral shafts	-40°C	-80°C
Clavert, 2001	France	1	Cadaveric	NR	NR	Tendons of the long head of the biceps brachii.	Saline, -30°C	Saline, -30°C
Hernigou, 2000	France	1	NR	NR	NR	Bone- Tibiae, femurs	NR	NR
Moreau, 2000	France	NR	NR	NR	NR	Bone- Femoral heads	NR	-30°C, then RT after defatting.

Table 3: Clinical Study Characteristics

<u>Quality of evidence</u>

Level I evidence: High

Level II evidence: Moderate

Level III evidence: Low

Level IV evidence: Very low

First author, Year	Country	Number of Sites	Clinical Study Type	Donor	Recovery Site	Pre-recovery sanitization (environment and donor)	Amount and Type of Recovered Tissue	Post Recovery Storage Conditions (short term)	Preservation Method
Sun, 2012	China	1	Single-blinded, prospective, randomized, clinical trial (Level I)	NR	NR	NR	Hamstring tendon	NR	Fresh-frozen
Sun, 2009	China	1	Prospective, randomized, clinical trial (Level I)	NR	NR	NR	Bone-patellar tendon-bone	NR	Fresh-frozen
Indelicato, 2013	United States	3	Randomized trial (Level II)	NR	NR	NR	Bone-patellar tendon-bone	NR	NR
Kim, 2011	South Korea	1	Prospective cohort study (Level II)	Cadaveric	NR	NR	Bone (iliac bone)& soft tissue (fascia)	NR	-70°C
Gajiwala, 2003	India	1	Prospective cohort (Level II)	Cadaveric or living	NR	NR	Bone- iliac crest, femoral head or long bone segments	NR	NR

First author, Year	Country	Number of Sites	Clinical Study Type	Donor	Recovery Site	Pre-recovery sanitization (environment and donor)	Amount and Type of Recovered Tissue	Post Recovery Storage Conditions (short term)	Preservation Method
Krasny, 2013	Poland	1	Retrospective chart review (Level III)	Cadaveric	NR	NR	Corticospongious bone blocks from the iliac ala, demineralized bone matrix from the compact bone of diaphysis	NR	NR
Guo, 2012	China	1	Retrospective cohort (Level III)	Living (self) and cadaveric	Operating theatre for autografts, sterilized bank site for allografts	Allografts harvested "sterilely" at bank	Bone-patellar tendon-bone	Refrigeration	Allografts: -30°C to -35°C, Autografts: no storage
Mehendale, 2009	United Kingdom	2	Retrospective chart review (Level III)	NR	NR	NR	Bone (femoral heads)	NR	NR
Galia, 2009	Brazil	1	Retrospective cohort study (Level III)	NR	NR	NR	Bone-morselized	NR	NR
Khoo, 2006	Australia	NR	Retrospective cohort study (Level III)	NR	NR	Allografts harvested under "sterile conditions".	Bones-femoral shafts, proximal humerus, femur, tibia, distal femur	NR	-70°C
Pruss	Germany	2	Retrospective	Cadaveric	Morgue or	"Clean	Cancellous	NR	Freeze-drying

First author, Year	Country	Number of Sites	Clinical Study Type	Donor	Recovery Site	Pre-recovery sanitization (environment and donor)	Amount and Type of Recovered Tissue	Post Recovery Storage Conditions (short term)	Preservation Method
Perka, 2002			cross-sectional study (Level III)		institute for pathology	conditions"	bone, cortical bone, amnion, demineralized bone matrix, ligament/tendon and others		or cryopreservation
