# Supplementary material for: Disinfection of human musculoskeletal allografts in tissue banking: a systematic review
Source: Cell Tissue Bank. 2016 Sep 24;17(4):573–84. doi: 10.1007/s10561-016-9584-3 (PMC5116033; doi:10.1007/s10561-016-9584-3)
Supplement: Supplementary file 5 — Supplementary material 5 (PDF 105 kb) [file 10561_2016_9584_MOESM5_ESM.pdf]

Appendix E

Table 4: Microbial Sampling Method for Laboratory Studies

First author, Year	Organisms Tested	Sample Preparation	Microbial Testing Method (Culturing, Serological, Nucleic Acid ID)[1]	Media type	Incubation Conditions
Elenes, 2014	NR	NR	NR	NR	NR
Kaminski, 2012	NR	NR	NR; Serology performed, specifics NR	NR	NR
Schubert, 2012	Bacteria	NR	Culturing	Thioglycolate broth culture medium or blood agar and chocolate plates under aerobic and anaerobic conditions	7 days
Hernandez, 2012	NR	NR	NR	NR	NR
Shaw, 2012	NR	NR	NR	NR	NR
Jung, 2011	NR	NR	NR	NR	NR
Schmidt, 2012	Viruses	After sterilization, the samples were stored and transported in the polystyrene boxes with dry ice at –78 °C to the virological laboratory and stored at –80 °C until virus titration was per- formed.	Culturing	Serial dilutions inoculated into 96 well-plate where cells were preseeded for one day.	37°C (5% CO_2, saturated H_2O atmosphere)
Cornu, 2011	NR	NR	NR	NR	NR
Hoburg 2011	NR	NR	NR	NR	NR
Barth, 2011	NR	NR	NR	NR	NR
Nguyen, 2011	Bacteria	Thawed bone samples were	Culturing	Trypticase soy agar	"Using standard

First author, Year	Organisms Tested	Sample Preparation	Microbial Testing Method (Culturing, Serological, Nucleic Acid ID)[1]	Media type	Incubation Conditions
	Fungi	rinsed with warm sterile saline and the solution was removed and filtered.		plates	microbiology protocols"
Reid, 2010	NR	NR	NR	NR	NR
Ketonis, 2010	Bacteria	Sterilized with 70% ethanol (15 min), washed 3x with PBS and 3x with TSB. *S. aureus* were incubated with the sterilized samples in TSB, 37°C, under static conditions for 6 to 24 h.	Cultured	Plated on 3 M1 Petri-films.	37°C, under static conditions for 6 hours in TSB; then incubated overnight
Hoburg, 2010	NR	NR	NR	NR	NR
Bitar, 2010	NR	NR	NR	NR	NR
Kaminski, 2009	NR	NR	NR	NR	NR
Vastel, 2009	NR	NR	NR	NR	NR
Saegeman, 2009	Bacteria	Bone fragments were shaken separately in sterile PBS for the antibiotic-treated fragments and in neutralisation solution for the antiseptic-treated fragments	Culturing	Tryptic Soy Agar Confirmatory test: Wilkins-Chalgren anaerobic broth, subcultured on blood agar plate.	Main test: NR, Confirmatory test: 36.5C, 7 d
Balsly, 2008	NR	NR	NR	NR	NR
Mikhael, 2008	NR	NR	NR	NR	NR
Parker, 2008	Bacteria	Bacteria on the orthopedic operating room floor were cultured.	Culturing	Environment: RODAC surface contact culture plates, Contaminated then treated samples: blood agar plates.	37°C for 10 days in aerobic conditions

First author, Year	Organisms Tested	Sample Preparation	Microbial Testing Method (Culturing, Serological, Nucleic Acid ID)[1]	Media type	Incubation Conditions
Kattaya, 2008	Bacteria	Mini spore strips were removed from the bones under aseptic conditions	Culturing	Tryptic soy broth media	35°C, 14 d
Nguyen, 2008	Bacteria	Warm Ringer's solution added to allograft, shake 5 min, then filter solution (0.45 um).	Culturing	Horse blood agar plate	37°C for 7 days
Haimi, 2008	NR	NR	NR; donors underwent “bacteriological and viral screening”	NR	NR
Han, 2008	NR	NR	NR	NR	NR
Giannini, 2008	NR	NR	NR	NR	NR
Henson, 2008	NR	NR	NR	NR	NR
Lewis, 2008	NR	NR	NR	NR	NR
Scheffler, 2007	Viruses	Samples were centrifuged. The supernatant was used for virus titre. The pelleted tissue was resuspended in DMEM and used for virus titration.	Cell culturing (PRV was titrated on BHK21 and PPV on pk13 cells); Donors ”tested for HBsAg, antibodies against HIV-1/2, HBc, HCV and TPHA and for HIV, HBVand HCV genome”	NR	3 or 7 days
Vastel, 2007	NR	NR	NR	NR	NR
Hilmy, 2007	Bacteria	Swabs of bone and membrane filtration for lyophilized an demineralized samples	Culturing; Serological testing for HIV, hepatitis B and C	Trypticase soy agar	30°C for 14 days
Jones, 2007	NR	NR	NR	NR	NR
Schimizzi, 2007	NR	NR	NR	NR	NR
Bienek, 2007	Virus	NR for bacteriophage, Spore	Culturing; Serology	Virus- Plaque assay	Virus- 37°C

First author, Year	Organisms Tested	Sample Preparation	Microbial Testing Method (Culturing, Serological, Nucleic Acid ID)[1]	Media type	Incubation Conditions
	(bacteriophage) and bacteria	strips from femoral heads sonicated in 10 ml tryptone soya broth	performed, specifics NR	with bacterial agar plates and E. coli; Bacterial spores-sonication in tryptone soya broth and inoculation of agar plates	overnight; Bacteria-22°C overnight
Grieb, 2006	Bacteria and viruses	Subset of tendons were inoculated with *Clostridium sordellii*, sterilized, and then Reinforced Clostridial medium was added to tendon and vortexed. Subset of tendons were inoculated with porcine parvovirus or Sindbis virus, sterilized, and then PBS with 0.5% FBS was added to tendon and vortexed.	Culturing<br><br>PBS/FBS viral content quantified with TCID50 assay (PVV with PK13 cells; Sindbis with Vero cells).	Blood agar plates.	Anaerobic culture 48 h
Mroz, 2006	NR	NR	NR	NR	NR
Akkus, 2005 (1)	NR	NR	NR	NR	NR
Akkus, 2005 (2)	NR	NR	NR	NR	NR
Scheffler, 2005	NR	NR	NR	NR	NR
Grieb, 2005	Bacteria, fungi and viruses	Media added to sample, vortexed for 30 seconds and centrifuged to pellet debris	Culturing	"Agar plates suitable for each microorganism or plaque assay for viruses"	7-10 days
Baker, 2005	Bacterias	Allograft immersed in growth media	Culturing	Soybean casein digest broth	30°C for 14 days

First author, Year	Organisms Tested	Sample Preparation	Microbial Testing Method (Culturing, Serological, Nucleic Acid ID)[1]	Media type	Incubation Conditions
Mitchell, 2004	NR	NR	NR	NR	NR
Lomas, 2004	NR	NR	NR; Serology performed, specifics NR	NR	NR
Moore, 2004	Viruses	"assayed for infectivity"	NR	NR	NR
Vastel, 2004	Bacteria	After contamination, small bone pieces or swabs	Culturing	Testing with acridine orange: positive samples were cultured aerobically on blood and chocolate agar, and anaerobically on fastidious anaerobic agar.	NR
Pruss,Kao et al 2003	Viruses	Samples from viral suspensions inside bone	Viral cell culture Human immunodeficiency virus type 2- Molt 4 clone 8	RPMI 1640	NR
			Viral cell culture Bovine viral diarrhoea virus- foetal calf cells	DMEM	
			Viral cell culture Pseudorabies virus in mink lung cells	DMEM	
			Viral cell culture Hepatitis A virus- fetal rhesus monkey kidney	DMEM	

First author, Year	Organisms Tested	Sample Preparation	Microbial Testing Method (Culturing, Serological, Nucleic Acid ID)[1]	Media type	Incubation Conditions
			Viral cell culture<br>Polio virus type 1 in human lung cells	DMEM	
			Viral cell culture<br>Bovine parvovirus in calf lung cells	DMEM	
			Serological testing for HIV-1 and -2, hepatitis B and C, TPHA		
Dunsmuir, 2003	Bacteria	Bone tissue cultured in Schaedler's broth	Culturing; Serological testing for HIV-1 and -2, hepatitis B and C and *T. pallidum*	Schaedler's broth cultures grown on Columbia blood agar plates	37°C for 24 h (aerobically)
Pruss,Gobel et al 2003	Bacteria, fungi and viruses	Viruses- samples from viral suspensions, bone incubation media, homogenized bone supernatant	Human immunodeficiency virus type 2-in lymphoma cells	RPMI 1640	10-14 d
			Bovine viral diarrhoea virus in calf lung cells	DMEM with anti-BVDV-free serum	3 d
			Pseudorabies virus in mink lung cells	DMEM	3 d
			Hepatitis A virus in fetal rhesus monkey kidney	DMEM	10-14 d
			Poliomyelitis virus type 1 in fetal lung cells	DMEM	5

First author, Year	Organisms Tested	Sample Preparation	Microbial Testing Method (Culturing, Serological, Nucleic Acid ID)[1]	Media type	Incubation Conditions
			Pocine parvovirus in fetal porcine testis cells	DMEM	7 d
			Culturing	Casein soy peptone agar-*S. aureus*, *P. aeruginosa*, *B. subtilis*, *C. sporogenes*	37°C for 48 h
		Bacteria and fungi- homogenized bone supernatant	Culturing	Casein soy peptone agar- *B. subtilis* spores	37°C for 48-72 h
			Culturing	Kanamycin-Esculin agar- *E. faecium*	37°C for 48 h
			Culturing	Middlebrook 7H10 Agar+OADC- *M. terrae*	37°C for 4 weeks
			Culturing	Sabouraud-glucose agar- *C. albicans*	37°C for 48 h
			Culturing	Sabouraud-glucose agar- *A. niger* spores	30°C for 48-72 h
			Serological testing for HIV-1 and -2, hepatitis B and C, TPHA, and PCR for HIV, hepatitis B and C		
Dufrane, 2002	NR	NR	NR; Serological testing for HIV-1 and -2, hepatitis B and C, HTLV 1,	NR	NR

First author, Year	Organisms Tested	Sample Preparation	Microbial Testing Method (Culturing, Serological, Nucleic Acid ID)[1]	Media type	Incubation Conditions
			syphilis		
Pruss, Kao *et al*, 2002	Viruses	Samples from viral suspensions alone and inside bone	Human immunodeficiency virus type 2-in lymphoma cells	RPMI 1640	37°C, 5% CO2
			Bovine viral diarrhoea virus in calf lung cells	DMEM with anti-BVDV-free serum	37°C, 5% CO2
			Pseudorabies virus in mink lung cells	DMEM	37°C, 5% CO2
			Hepatitis A virus in fetal rhesus monkey kidney	DMEM	37°C, 5% CO2
			Poliomyelitis virus type 1 in fetal lung cells	DMEM	37°C, 5% CO2
			Pocine parvovirus in fetal porcine testis cells	DMEM	37°C, 5% CO2
			Serological testing for HIV-1 and -2, hepatitis B and C and *T. pallidu*, and PCR for HIV, hepatitis B and C		
Pruss, 2001	Bacteria, fungi	Sanitization supernatant and homogenized bone samples	Culturing; Serological testing for HIV-1 and -2, hepatitis B and C, TPHA	Casein soy peptone agar-*S. aureus, P. aeruginosa, B. subtilis, C.*	NR

First author, Year	Organisms Tested	Sample Preparation	Microbial Testing Method (Culturing, Serological, Nucleic Acid ID)[1]	Media type	Incubation Conditions
				sporogenes	
				Casein soy peptone agar- *B. subtilis* spores	
				Kanamycin-Esculin agar- *E. faecium*	
				Middlebrook 7H10 Agar+OADC- *M. terrae*	
				Sabouraud-glucose agar- *C. albicans*	
				Sabouraud-glucose agar- *A. niger* spores	
Akkus, 2001	NR	NR	NR	NR	NR
Lomas, 2001	NR	NR	NR	NR	NR
Clavert, 2001	NR	NR	NR	NR	NR
Hernigou, 2000	Virus	Dilutions of viruses cultured in MT2 cells	Microscopy	Inverted phase contrast microscope visualized viral replication in MT2 cells	37°C, 5% CO2 for up to 1 week

[1]Serological analysis includes antibody and antigen using ELISAs or other methods. Nucleic acid Identification can be for RNA or DNA.

Table 6: Microbial Sampling Method for Clinical Studies

First author, Year	Organisms Tested	Sample Preparation	Microbial Testing Method (Culturing, Serological, Nucleic Acid ID)[1]	Media type	Incubation Conditions

First author, Year	Organisms Tested	Sample Preparation	Microbial Testing Method (Culturing, Serological, Nucleic Acid ID)[1]	Media type	Incubation Conditions
Sun, 2012	NR	NR	NR	NR	NR
Sun, 2009	NR	NR	NR; Serology performed, specifics NR	NR	NR
Indelicato, 2013	NR	NR	NR	NR	NR
Kim, 2011	NR	NR	NR; serological testing for HIV, hepatitis B and C, cytomegalovirus, syphilis	NR	NR
Gajiwala, 2003	NR	Swab cultures	NR; serological testing for HIV, hepatitis B and C, syphilis	NR	NR
Krasny, 2013	NR	NR	NR; Serology performed, specifics NR	NR	NR
Guo, 2012	NR	NR	NR	NR	NR
Mehendale, 2009	NR	NR	NR	NR	NR
Galia, 2009	NR	NR	NR	NR	NR
Khoo, 2006	Bacteria	Wound swabs, intra-operative specimens, aspirated fluid for post-transplantation results	NR	NR	NR
Pruss Perka, 2002	Virus	NR	NR; donors “clinically examined” for AIDS, hepatitis virus, tuberculosis, Lues, septicaemia, systemic viral disease and mycosis	NR	NR

[1]Serological analysis includes antibody and antigen using ELISAs or other methods. Nucleic acid Identification can be for RNA or DNA.
