# Supplementary material for: Disinfection of human musculoskeletal allografts in tissue banking: a systematic review
Source: Cell Tissue Bank. 2016 Sep 24;17(4):573–84. doi: 10.1007/s10561-016-9584-3 (PMC5116033; doi:10.1007/s10561-016-9584-3)
Supplement: Supplementary file 6 — Supplementary material 6 (PDF 369 kb) [file 10561_2016_9584_MOESM6_ESM.pdf]

Appendix F

Table 7: Recovery and Processing of Allografts for Laboratory Studies

First Author, Year	Tissue Recovered	Number of samples	Cleaning of Tissue	Proportion of contamina-tion following recovery	Isolated Micro-organisms	Sanitization Method	Sanitization Parameters	Tissue Integrity	Proportion of positive cultures or microbial load	Logarithmic bioburden reduction
Elenes, 2014	Tendons-bone-patellar tendon-bone and tibialis tendons<br>Bone (femur)<br>Bone and tendon	80 tendons<br>20 donors	NR	NR	NR	17.1 to 21 kGy gamma radiation;	NR	E-beam or gamma radiation did not change any of the mechanical properties tested.	NR	NR
						9.2 to 12.2 kGy electron beam				
						17.1 to 21 kGy electron beam				
Kaminski, 2012	Bone (femur)	48 samples<br>6 donors	Femurs were mechanically cleaned of soft tissues and stored at -70 °C. Epiphyses of the frozen femurs were cut off and stored at -70 °C for future experiments.	NR	NR	Accelerated electron beam irradiation (25 kGy)	NR	No change	NR	NR
						Accelerated electron beam irradiation (35 kGy)		Increase in resilience and elastic limit		
Schubert, 2012	Bone and tendon	474 donors	Cleaning and isolation done but details are NR	10.1% positive culture (365/3612)	*Coagulase neg. Staphylococcus,*	No treatment		NR	18.8% (39/208)	NR

First Author, Year	Tissue Recovered	Number of samples	Cleaning of Tissue	Proportion of contamina-tion following recovery	Isolated Micro-organisms	Sanitization Method	Sanitization Parameters	Tissue Integrity	Proportion of positive cultures or microbial load	Logarithmic bioburden reduction
					Micrococcus, Bacillus cereus, Corynebacterium, Penicilium, Alcaligenes, Lactobacillus, Eschericcia coli, Acinetobacter, Enterococcus, Pseudomonas aeruginosa, Pneumococcus, Neisseria, Candida	0.1% thimerosal	1 hour		8% (42/527)	
						1.2 g/l rifampicin	1 hour		4.6% (79/1712)	
						No antiseptics			6.9% (7/101)	
						90% alcohol	1 hour		10.4% (12/115)	
						0.02% chlor-hexidine in 70% alcohol	1 hour		4.6% (143/3099)	
Hernandez, 2012	Bone (femurs)	17 samples 10 donors	Removed marrow from specimens with a low-pressure water jet, ends of the specimen were polished to	NR	NR	Gamma irradiation (30kGy),	4 hours	No difference in Young's modulus, yield strain, yield stress, and residual strain; no	NR	NR

First Author, Year	Tissue Recovered	Number of samples	Cleaning of Tissue	Proportion of contamina-tion following recovery	Isolated Micro-organisms	Sanitization Method	Sanitization Parameters	Tissue Integrity	Proportion of positive cultures or microbial load	Logarithmic bioburden reduction
			ensure parallel alignment,					differences in microcrack density, dif-fuse damage, or trabecular microfracture between irradiated and control groups		
Schmidt, 2012	"principles of a tendon model"	NR	NR	100% (inoculated with viruses)	HIV-2<br>Pseudorabies virus (PRV)<br>HAV<br>PPV	Fractionated Electron Beam irradiation	NR	NR	Both methods reveal comparable and sufficient virus inactivation capacities.	Fractionated >4.0
						Standard Electron Beam irradiation				Standard >4.0

First Author, Year	Tissue Recovered	Number of samples	Cleaning of Tissue	Proportion of contamina-tion following recovery	Isolated Micro-organisms	Sanitization Method	Sanitization Parameters	Tissue Integrity	Proportion of positive cultures or microbial load	Logarithmic bioburden reduction
Shaw, 2012	Bone- fibulae	8 donors	Debrided	NR	NR	Allowash®	NR	No impact of freeze-thaw cycles (up to 8) on mechanical properties. Freeze-drying increased both stiffness (53%) and energy to failure (117%).	NR	NR
Jung, 2011	Patella, patellar tendon and at least 10 cm of tibial bone	21 donors	NR	NR	NR	None performed	None	The number of freeze-thaw cycles had no impact on the mechanical properties of the tendon (creep, stiffness, ultimate load, tangent modulus)	NR	NR
Cornu, 2011	Bone (femurs)	36 samples 18 donors	One treatment underwent washing, solvent-detergents,	NR	NR	Gamma irradiation (25 kGy) WITHOUT freeze-drying,	Irradiation: 3 h under dry ice	No difference in ultimate stress, stiffness, work to failure.	NR	NR

First Author, Year	Tissue Recovered	Number of samples	Cleaning of Tissue	Proportion of contamination following recovery	Isolated Micro-organisms	Sanitization Method	Sanitization Parameters	Tissue Integrity	Proportion of positive cultures or microbial load	Logarithmic bioburden reduction
			proteins denaturation (prion inactivating procedure) and rinsing with deionized water			Gamma irradiation (25 kGy) then freeze-dried	Irradiation: 3 h under dry ice; freeze-drying process: 50 h.	As a percentage of the control (no treatment). Ultimate stress: -31.8 ($p<0.001$); stiffness: -37.92 ($p<0.001$); work to failure: -38.30 ($p<0.001$)		
						Chemical treatment then gamma irradiation (25 kGy) and freeze-dried control (frozen only)	Irradiation: 3 h under dry ice; freeze-drying process: 50 h.	As a percentage of the control (no treatment). Ultimate stress: -33.85 ($p<0.001$); stifness: -14.59 ($p<0.01$); work to failure: -44.17 ($p<0.001$)		
Hoburg 2011	Bone-patellar tendon-bone	44 samples	NR	NR	NR	Electron beam irradiation (34 kGy)	30 seconds	Gamma radiation had the greatest	NR	NR

First Author, Year	Tissue Recovered	Number of samples	Cleaning of Tissue	Proportion of contamina-tion following recovery	Isolated Micro-organisms	Sanitization Method	Sanitization Parameters	Tissue Integrity	Proportion of positive cultures or microbial load	Logarithmic bioburden reduction
						Fractionated electron beam irradiation (34 kGy)	average 17.5 hours	negative impacts on mechanical properties (strain, cyclic elongation response, failure load, stiffness). The Ebeam fraction had less of a negative impact than Ebeam.		
						Gamma irradiation (34 kGy)	11.8 minutes			
Barth, 2011	Bone (femur)	89 samples	NR	NR	NR	X-ray irradiation 0.05 kGy	NR	For irradiation exposures starting between 35 and 70 kGy, there is a severe dose-dependent degradation in mechanical properties; no effect could be discerned at 35 kGy and below	NR	NR
						25 kGy				
						35 kGy				
						70 kGy				
						210 kGy				
						630 kGy				

First Author, Year	Tissue Recovered	Number of samples	Cleaning of Tissue	Proportion of contamina-tion following recovery	Isolated Micro-organisms	Sanitization Method	Sanitization Parameters	Tissue Integrity	Proportion of positive cultures or microbial load	Logarithmic bioburden reduction
Nguyen, 2011	Bone (femoral heads; structure and morselized allografts)	200 samples	NR	0%	NR	Gamma irradiation (average: 1.22-1.28 kGy;; required dose: 1.3 kGy)- RSD was based on average bioburden and then selected a verification dose from a database that gives the sterility assurance level (SAL)for the average bioburden	NR	NR	Bioburden: 0/10 (0%) of frozen bome allografts; all bone segments were negative (sterile) (0/200 (0%))	NR
Reid, 2010	Bone-patellar tendon-bone	42 tendons	NR	NR	NR	Aseptic harvest with low-dose radiation processing	NR	No statistical differences in the mechanical outcomes: 2% yield stress	NR	NR

First Author, Year	Tissue Recovered	Number of samples	Cleaning of Tissue	Proportion of contamina-tion following recovery	Isolated Micro-organisms	Sanitization Method	Sanitization Parameters	Tissue Integrity	Proportion of positive cultures or microbial load	Logarithmic bioburden reduction
						BioCleanse™ Tissue Processing System		(MPa), Young's modulus (MPa), elongation failure (mm), strain fracture (%), ultimate stress (MPa), and toughness (kJ)		
						Clearant® Process				
Ketonis, 2010	Bone (cortical stubs or morselized bone)	NR	Cortical bone was cut into 1 x 1-cm squares, washed, and	100% positive culture (21/21)	*S. aureus*	None- trypticase soy broth (TSB)	16 h,<br>4 h after inoculation<br>12 hours	NR	NR	0.50

First Author, Year	Tissue Recovered	Number of samples	Cleaning of Tissue	Proportion of contamina-tion following recovery	Isolated Micro-organisms	Sanitization Method	Sanitization Parameters	Tissue Integrity	Proportion of positive cultures or microbial load	Logarithmic bioburden reduction
			sonicated with dH2O. Samples were partially demineralized. Before synthesis, all samples were washed with dH2O and sonicated twice for 30 min in dimeth ylformamid; Bone stubs were coupled twice with 10 mg/mL Fmoc-[2-(2-amino-ethoxy)-ethoxy]-acetic acid followed by chemical coupling with 10 mg/ mL clinical grade vancomycin; then washed with DMF	(inoculated by researchers)		TSB + 10 ug/mL VAN	during inoculation		NR	4.5

First Author, Year	Tissue Recovered	Number of samples	Cleaning of Tissue	Proportion of contamina-tion following recovery	Isolated Micro-organisms	Sanitization Method	Sanitization Parameters	Tissue Integrity	Proportion of positive cultures or microbial load	Logarithmic bioburden reduction
Hoburg, 2010	Bone-patellar tendon-bone	32 samples from 10 donors	NR	NR	NR	Electron beam irradiation 15 kGy	average: 30 seconds	No differences in strain behavior between the nonsterilized (4.02, 0.83) and the sterilized grafts with 15 kGy (3.28, 0.96), 25 kGy (4.02, 1.12), or 34 KGy (4.29,0.79; P=0.342); no differences in cyclic elongation; no effect on stiffness and failure load accept for irradiation with 34 kGy, which caused a significantly lower failure load than control	NR	NR
						Electron beam irradiation 25 kGy	average: 30 seconds			
						Electron beam irradiation 34 kGy	average: 30 seconds			

First Author, Year	Tissue Recovered	Number of samples	Cleaning of Tissue	Proportion of contamina-tion following recovery	Isolated Micro-organisms	Sanitization Method	Sanitization Parameters	Tissue Integrity	Proportion of positive cultures or microbial load	Logarithmic bioburden reduction
Bitar, 2010	Semi-tendinous muscle tendons	20 tendons 10 donors	NR	NR	NR	NR	NR	Histological analysis observed no differences between fresh and frozen allografts.	NR	NR
Kaminski, 2009	Bone-patellar tendon-bone	50 samples from 25 donors	NR	NR	NR	Irradiation 25 kGy	Dry Ice	Tensile strength-irradiated group have approx 20% lower compared to their controls (non-irradiated); no differences between the different doses (25-100kGy); tensile strength decrease was greater with lyophilisation or glycerolisation	NR	NR
						35 kGy	Dry Ice			
						50 kGy	Dry Ice			
						100 kGy	Dry Ice			
						35 kGy	Room temperature			
						35 kGy	Dry ice			
						35 kGy	Room temperature			

First Author, Year	Tissue Recovered	Number of samples	Cleaning of Tissue	Proportion of contamina-tion following recovery	Isolated Micro-organisms	Sanitization Method	Sanitization Parameters	Tissue Integrity	Proportion of positive cultures or microbial load	Logarithmic bioburden reduction
Vastel, 2009	Bone (femoral heads)	14 donors	Irrigation with saline	NR	NR	-TBF (sodium azide, ethanol-chloroform, H_2O_2, atm. Pressure, delipidation-sterilization, hypochlorite, atm. Pressure, delipidation-sterilization, sublimation-drying, irradiation with 25 kGy),	12 hours in sodium azide, 19 hours in ethanol-chloroform,19 hours in H_2O_2, 2 hours in hypochlorite, atm	Bone mechanical properties, as assessed by ultrasound velocity-induced a statistically significant ($p < 0.05$) change that ranged from 7.8% to 10.2% according to the axis and frequency.	NR	NR
						Moist heat	20 minutes at 125°C<br>>48 hours	Induced nonsignificant changes at low frequency measurement and small but significant changes at high frequency ($p < 0.05$), which ranged from 1.3% to 2.2%.		

First Author, Year	Tissue Recovered	Number of samples	Cleaning of Tissue	Proportion of contamina-tion following recovery	Isolated Micro-organisms	Sanitization Method	Sanitization Parameters	Tissue Integrity	Proportion of positive cultures or microbial load	Logarithmic bioburden reduction
						Tutoplast (successive baths of physiological saline with osmotic detersion, rinsing drying and 18kGy irradiation)		Induced a significant change ($p < 0.05$) ranging from 2.5% to 5.7%		
						Supercrit (CO_2 in the supercritical phase, delipidation-sterilization, H2O2, NaOH, ethanol, Atm. pressure, delipidation sterilization, Drying, Atm pressure, 25 kGy irradiation)	CO_2 in the supercritical phase- 50°C; timeframe: NR	Induced nonsignificant changes at low frequency measurement and small but significant changes at high frequency ($p < 0.05$), which ranged from 1.3% to 2.2%.		

First Author, Year	Tissue Recovered	Number of samples	Cleaning of Tissue	Proportion of contamina-tion following recovery	Isolated Micro-organisms	Sanitization Method	Sanitization Parameters	Tissue Integrity	Proportion of positive cultures or microbial load	Logarithmic bioburden reduction
Saegeman, 2009	Bone	NR	Soft tissue as removed	100% positive culture (inoculated by researchers) (after rinsed, decontaminated in ethanol 75% for 30 min and gamma-irradiated (25 kGy) to ensure sterility)	*Staphylococcus haemolyticus S. epidermidis*	Gentamicin (512 µg/mL) + (rinsed, decontaminated in ethanol 75% for 30 min and gamma-irradiated (25 kGy) after recovery)	18 h at 36.5°C and at 24°C (room temperature).	NR	# of positive broth: 7 total (for 4 different strains)	NR
						Gentamicin (1,024 µg/mL) + (rinsed, decontaminated in ethanol 75% for 30 min and gamma-irradiated (25 kGy) after recovery)	18 h at 36.5°C and at 24°C (room temperature).		# of positive broth: 8 total (for 4 different strains)	

First Author, Year	Tissue Recovered	Number of samples	Cleaning of Tissue	Proportion of contamina-tion following recovery	Isolated Micro-organisms	Sanitization Method	Sanitization Parameters	Tissue Integrity	Proportion of positive cultures or microbial load	Logarithmic bioburden reduction
						Rifampicin (400 µg/mL) + (rinsed, decontaminated in ethanol 75% for 30 min and gamma-irradiated (25 kGy) after recovery)	18 h at 36.5°C and at 24°C (room temperature).		# of positive broth: 16 total (for 7 various test strains of gentamicin and rifapicin susceptibility)	
						Rifampicin (1,000 µg/mL) + (rinsed, decontaminated in ethanol 75% for 30 min and gamma-irradiated (25 kGy) after recovery)	18 h at 36.5°C and at 24°C (room temperature).		# of positive broth: 9 total (for 7 various test strains of gentamicin and rifapicin susceptibility)	
						0.5% chlor-hexidine in 70% alcohol + (rinsed, decontaminated in ethanol	NR		# of positive broth: 12 total (for 7 various test strains of	

First Author, Year	Tissue Recovered	Number of samples	Cleaning of Tissue	Proportion of contamina-tion following recovery	Isolated Micro-organisms	Sanitization Method	Sanitization Parameters	Tissue Integrity	Proportion of positive cultures or microbial load	Logarithmic bioburden reduction
						75% for 30 min and gamma-irradiated (25 kGy) after recovery			gentamicin and rifapicin susceptibility)	
						4% chlorhexidine soap + (rinsed, decontaminated in ethanol 75% for 30 min and gamma-irradiated (25 kGy) after recovery)	NR		# of positive broth: 9 total (for 7 various test strains of gentamicin and rifapicin susceptibility)	
						Gentamicin (512 µg/mL) + 60 min drying+ (rinsed, decontaminated in ethanol 75% for 30 min and gamma-irradiated (25 kGy) after recovery)	18 h at 36.5°C and at 24°C (room temperature).		# of positive broth: 7 total (for 4 different strains)	

First Author, Year	Tissue Recovered	Number of samples	Cleaning of Tissue	Proportion of contamina-tion following recovery	Isolated Micro-organisms	Sanitization Method	Sanitization Parameters	Tissue Integrity	Proportion of positive cultures or microbial load	Logarithmic bioburden reduction
						Gentamicin (1024 µg/mL) + 60 min drying+ (rinsed, decontaminated in ethanol 75% for 30 min and gamma-irradiated (25 kGy) after recovery)	18 h at 36.5°C and at 24°C (room temperature).		# of positive broth: 8 total (for 3 different strains)	
						Rifampicin (400 µg/mL) (1024 µg/mL) + 60 min drying+ (rinsed, decontaminated in ethanol 75% for 30 min and gamma-irradiated (25 kGy) after recovery)	18 h at 36.5°C and at 24°C (room temperature).		# of positive broth: 7 total (for 4 different strains)	

First Author, Year	Tissue Recovered	Number of samples	Cleaning of Tissue	Proportion of contamina-tion following recovery	Isolated Micro-organisms	Sanitization Method	Sanitization Parameters	Tissue Integrity	Proportion of positive cultures or microbial load	Logarithmic bioburden reduction
						Rifampicin (1000 µg/mL) (1024 µg/mL) + 60 min drying+ (rinsed, decontaminated in ethanol 75% for 30 min and gamma-irradiated (25 kGy) after recovery)	18 h at 36.5°C and at 24°C (room temperature).		# of positive broth: 9 total (for 7 different strains)	
Balsly, 2008	Bone (cloward dowels and iliac crest wedges) and soft tissue (patellar tendons, anterior tibialis tendons, semitendinosus tendons, and	NR	Bone dowels and iliac crest wedges were debrided	NR	NR	Irradiation (18.3–21.8 kGy- lose dose)	Dry ice at temperature of -20°C to -50°C for approximately 2–3 h	no effect on the strength and elastic modulus of any allograft (compared to control);	NR	NR

First Author, Year	Tissue Recovered	Number of samples	Cleaning of Tissue	Proportion of contamina-tion following recovery	Isolated Micro-organisms	Sanitization Method	Sanitization Parameters	Tissue Integrity	Proportion of positive cultures or microbial load	Logarithmic bioburden reduction
	fascia lata)					Irradiation(24.0–28.5 kGy- moderate dose)	Dry ice at temperature of -20°C to -50°C for approximately 2–3 h	no effect on the strength and elastic modulus of bone allografts (compared to control) Patellar tendon demonstrated a loss in tensile strength, but its modulus of elasticity was unchanged		

First Author, Year	Tissue Recovered	Number of samples	Cleaning of Tissue	Proportion of contamina-tion following recovery	Isolated Micro-organisms	Sanitization Method	Sanitization Parameters	Tissue Integrity	Proportion of positive cultures or microbial load	Logarithmic bioburden reduction
Mikhael, 2008	Bone (femur or tibia)	7 donors	specimens for the axial, diametral, and shear test groups were obtained from the tibia, while the three-point bend specimens were obtained from the femur	NR	NR	BioCleanse (chemical sterilization)	NR	No significant difference in ultimate stress, ultimate strain, fracture energy, fracture displace-ment, or Young's modulus among the four treatment groups.<br><br>The Control, Chemical sterilization, and Chemical sterilization+ irradiation groups had significantly greater ultimate fracture energy, and fracture displacement	NR	NR

First Author, Year	Tissue Recovered	Number of samples	Cleaning of Tissue	Proportion of contamina-tion following recovery	Isolated Micro-organisms	Sanitization Method	Sanitization Parameters	Tissue Integrity	Proportion of positive cultures or microbial load	Logarithmic bioburden reduction
						BioCleanse + gamma irradiation (BioCleanses and terminal sterilization with exposure to approximately 20–23 kGy of gamma irradiation)		Axial compression: no statistically significant differences noted for ultimate strain or fracture displacement between the groups Diameteral compression decreased		
						Freeze dried (BioCleanses, lyophilization, terminal sterilization by STERRAD and rehydration)		Axial compression: Freeze dried group had a significantly greater ultimate compression stress, fracture energy, and Young's modulus than the Chemical sterilization alone Diameteral		

First Author, Year	Tissue Recovered	Number of samples	Cleaning of Tissue	Proportion of contamination following recovery	Isolated Micro-organisms	Sanitization Method	Sanitization Parameters	Tissue Integrity	Proportion of positive cultures or microbial load	Logarithmic bioburden reduction
								compression decreased		
Parker, 2008	Bone-patellar tendon-bone	12 samples from 6 donors	Specimens were divided into grafts measuring 8 mm in width. Specimens were prepared to a length of 70 mm (30-mm bone and 40-mm tendon) and placed into individual sterile specimen cups; Prior to	100% positive culture (40/40) (inoculated by researchers)	*Staphylococcus species*, *gram-negative bacilli*, *Bacillus species*, *diphtheroids*, *coagulase-positive Staphylococcus species*,	Control group (unsterilized)	NR	NR	CFU- Bone (Staphylococcus: 33.1; Bacillus: 10.5), Tendon (Staph: 31; Bacillus: 6.2), Combined graft (Staph: 63.1; Bacillus: 16.7)	Control-NR

First Author, Year	Tissue Recovered	Number of samples	Cleaning of Tissue	Proportion of contamina-tion following recovery	Isolated Micro-organisms	Sanitization Method	Sanitization Parameters	Tissue Integrity	Proportion of positive cultures or microbial load	Logarithmic bioburden reduction
			innoculation/contamination: sterilized with ethylene oxide in an Amsco Gas Sterilizer (4 hours at 38°C with 12% ethylene oxide at 8 to 10 PSI followed by 12 hours in an aerator at 54°C)		*and Micrococcus species*	Antibiotic Soak (polymyxin B, 166.66 units/cc and bacitracin, 16.66 units/cc)	15 minutes		Mean CFU- Bone (Staph: 7.6; Bacillus: 1.3), Tendon (Staph: 7.3; Bacillus: 1.2), Combined graft (Staph: 14.9; Bacillus: 2.5)	Staph 0.63, Bacillus 0.82
						Pulsatile Lavage (polymyxin B, 166.66 units/cc and bacitracin, 16.66 units/cc)	NR		Mean CFU- Bone (*Staph:* 0.2; *Bacillus*: 0), Tendon (*Staph:* 0.3; *Bacillus:* 0.1), Combined graft (*Staph:* 0.5; *Bacillus:* 0.1)	Staph 2.1, Bacillus 2.26

First Author, Year	Tissue Recovered	Number of samples	Cleaning of Tissue	Proportion of contamina-tion following recovery	Isolated Micro-organisms	Sanitization Method	Sanitization Parameters	Tissue Integrity	Proportion of positive cultures or microbial load	Logarithmic bioburden reduction
						Mechanical Agitation and Serial Dilution(poly myxin B, 166.66 units/cc and bacitracin, 16.66 units/cc)	2.5 minutes (Ten 15 second cycles)		Mean CFU- Bone (*Staph*: 0; *Bacillus*: 0), Tendon (*Staph*: 0; *Bacillus*: 0), Combined graft (*Staph*: 0; *Bacillus*: 0)	Agitation/dilution- Staph >1.80, Bacillus >1.2
Kattaya, 2008	Bone (femur)	NR	NR	embedded and sealed spore strips (1.1 x 10^6 spores/strip) in cortical bone segments	*Bacillus subtilis*	Control (in PBS only)	Solution: 24 hours at -4°C	NR	Not quantifiable - SEE FIGURES; Summary: L-cysteine s do not protect bacterial spores against gamma radiation and may be suitable for curbing the radiation	NR
						Gamma irradiation (0.64 Megarad or 6.4 kGy)	NR			
						L-cysteine	24 hours at -4°C			

First Author, Year	Tissue Recovered	Number of samples	Cleaning of Tissue	Proportion of contamina-tion following recovery	Isolated Micro-organisms	Sanitization Method	Sanitization Parameters	Tissue Integrity	Proportion of positive cultures or microbial load	Logarithmic bioburden reduction
						L-cysteine + irradiation (0.64 Megarad or 6.4 kGy)	24 hours at -4°C		damage to bone grafts while achieving sterility; spores were sensitized to radiation damage when irradiated in nitroimidazole-linked phenanthridinium solution and thus it is not an ideal radioprotectant	
						Nitroimidazole-linked-phenanthridinium	24 hours at -4°C			
						Nitroimidazole-linked-phenanthridinium + irradiation (0.64 Megarad or 6.4 kGy)	24 hours at -4°C			

First Author, Year	Tissue Recovered	Number of samples	Cleaning of Tissue	Proportion of contamina-tion following recovery	Isolated Micro-organisms	Sanitization Method	Sanitization Parameters	Tissue Integrity	Proportion of positive cultures or microbial load	Logarithmic bioburden reduction
Nguyen, 2008	Bone (femoral heads, structural bone and milled bone allografts)	80 samples from 2 donors	FEMORAL HEADS: all soft tissue, surface cartilage, and unusual calcified tissue removed, then bone segments were soaked in methanol 20 ml/l in ethanol 70% v/v for 10 min MILLED BONE: Cancellous bone was cut into small pieces. Fat tissue and bone marrow were washed using 0.9% sodium chloride solution for irrigation, bone	NR<br><br>Carryover experiment on femoral heads: 100% (9/9) (innoculate d with Bacillus pumilus (at 10^2 organisms/ 0.1 ml)	NR<br><br>Carryover experiment: *Bacillus subtilis*	Gamma irradiation (bioburdens for each bone allograft group were used to calculate the verification doses; 15 kGy was substantiated as radiation sterilisation dose)	NR	NR	0%	2 log reduction from inoculation (100 bact)

First Author, Year	Tissue Recovered	Number of samples	Cleaning of Tissue	Proportion of contamina-tion following recovery	Isolated Micro-organisms	Sanitization Method	Sanitization Parameters	Tissue Integrity	Proportion of positive cultures or microbial load	Logarithmic bioburden reduction
			was then soaked in 6.0% hydrogen peroxide w/v for 10 min, and then in 70% ethanol for 10 min; STRUCTURAL BONE: All soft tissue and surface cartilage were removed; bone marrow tissue in the canals was washed using a SurgicLav Plus Handpiece®, bones were cut then soaked into 70% ethanol for at least 30 min and dried			Hydrogen peroxide for 10 minutes, methylated spirit 70% for 10 minutes, dried for 5 minutes and washed for 60 mL	Approx 25 minutes		61% (58/95) spores detected after treatment	

First Author, Year	Tissue Recovered	Number of samples	Cleaning of Tissue	Proportion of contamina-tion following recovery	Isolated Micro-organisms	Sanitization Method	Sanitization Parameters	Tissue Integrity	Proportion of positive cultures or microbial load	Logarithmic bioburden reduction
Haimi, 2008	Bone (tibia and femur)	18 samples from 8 donors	Longitudinal cortical specimens were cut from the diaphys- ial part of the bones, using a grinding machine; The bone samples were continuously irrigated with distilled water throughout machining	NR	NR	Cleansing procedure A (sonication and four washes in distilled water, 3% hydrogen peroxide then 70% ethanol at room at room temperature, five washes in distilled water)	Four washes in distilled water- 90 min total at 60°C; wash in H2O2 and EtOH- 10 min, room temperature; additional five washes in distilled water (40 min total and overnight for 20 h) at 60°C	Mechanical testing: The bending strength and the absorbed energy of the processed cortical samples (cleansing procedures A to C) were increased slightly but the Young's	NR	NR

First Author, Year	Tissue Recovered	Number of samples	Cleaning of Tissue	Proportion of contamina-tion following recovery	Isolated Micro-organisms	Sanitization Method	Sanitization Parameters	Tissue Integrity	Proportion of positive cultures or microbial load	Logarithmic bioburden reduction
						Cleansing procedure B (modification of cleansing procedure A- but incubation times with hydrogen peroxide and 70% ethanol were doubled	-Four washes in distilled water- 90 min total at 60°C; wash in H2O2 and EtOH- 20 min, room temperature; additional five washes in distilled water (40 min total and overnight for 20 h) at 60°C	modulus was decreased compared to unprocessed samples		
						Cleansing procedure C (the overnight and two 10 minute washes in distilled were omitted)	Four washes in distilled water- 90 min total at 60°C; wash in H2O2 and EtOH- 10 min, room temperature; additional five washes in distilled water (20 min total) at 60°C			

First Author, Year	Tissue Recovered	Number of samples	Cleaning of Tissue	Proportion of contamina-tion following recovery	Isolated Micro-organisms	Sanitization Method	Sanitization Parameters	Tissue Integrity	Proportion of positive cultures or microbial load	Logarithmic bioburden reduction
Han, 2008	Bone	16 samples	DBM was made from donor cadaver bone by removing marrow, fat, and calcium while retaining extracellular components, mainly type I collagen and soluble growth factors and cytokines.	NR	NR	Gamma irradiation 0 kGy	NR	Gamma irradiation does not change cell attachment to the DBM matrix but has an influence on both stem cell and osteoprecursor cell proliferation rate; In Vitro Cell Attachment (in rats): no effect, collagen stability: 25-kGy-treated DBM became	NR	NR
						Gamma irradiation 12 kGy				
						Gamma irradiation 18 kGy				

First Author, Year	Tissue Recovered	Number of samples	Cleaning of Tissue	Proportion of contamina-tion following recovery	Isolated Micro-organisms	Sanitization Method	Sanitization Parameters	Tissue Integrity	Proportion of positive cultures or microbial load	Logarithmic bioburden reduction
						Gamma irradiation 25 kGy		more resistant to enzymatic digestion; In Vitro Osteoinductivity of Irradiated DBM: there was a dose-dependent decrease of ALP activity with increased irradiation dosages; mineral density associated with DBM appeared to decrease with the increase of the radiation dosage		
Giannini, 2008	Posterior tibial tendons	22 tendons from 11 donors	NR	NR	NR	None	NR	Freezing at -80°C caused decrease in ultimate load, ultimate stress,	NR	NR

First Author, Year	Tissue Recovered	Number of samples	Cleaning of Tissue	Proportion of contamina-tion following recovery	Isolated Micro-organisms	Sanitization Method	Sanitization Parameters	Tissue Integrity	Proportion of positive cultures or microbial load	Logarithmic bioburden reduction
								ultimate strain and increased stiffness. Freezing also resulted in increased diameter of collagen fibrils and decreased number of fibrils.		
Henson, 2008	Tibialis posterior tendon	4 tendons from 2 donors	NR	NR	NR	LifeCell and FlexACELL	2 h	Diameter, colour, stiffness, resilience: Tendons incubated for 8 h were smaller in diameter compared to 2 h, and were a	NR	NR

First Author, Year	Tissue Recovered	Number of samples	Cleaning of Tissue	Proportion of contamina-tion following recovery	Isolated Micro-organisms	Sanitization Method	Sanitization Parameters	Tissue Integrity	Proportion of positive cultures or microbial load	Logarithmic bioburden reduction
							8 h	different colour. Incubation time and rehydration time influenced stiffness, resilience and ease of handling (shorter cryoprotectant incubation and longer rehydration had better results.		
Lewis, 2008	Menisci	7 samples	NR	NR	NR	NR	NR	4 freeze-thaw cycles (-80°C to 25°C) decreased Young’s modulus of menisci, compared to 1 freeze-thaw cycle. Multiple freeze-thaw	NR	NR

First Author, Year	Tissue Recovered	Number of samples	Cleaning of Tissue	Proportion of contamina-tion following recovery	Isolated Micro-organisms	Sanitization Method	Sanitization Parameters	Tissue Integrity	Proportion of positive cultures or microbial load	Logarithmic bioburden reduction
								cycles also increased menisci water content.		
Scheffler, 2007	Soft tissue (Achilles tendon, skin and cartilage)	NR	Tissues were cut into small fragments. After rapid freezing in liquid nitrogen, tissues were ground to a particle size of 1-2 mm using an electric rotation mill; subsequently 1 cm3 of the granulated tissue was aseptically placed into a 15 ml Falcon tube and lyophilised	100% (mixed with a virus suspension (v/v 1 cm3/1 ml)	Enveloped pseudorabies virus (PRV, herpesviridae, dsDNA) and the non-enveloped porcine parvovirus (PPV, par-voviridae, ssDNA)	Peracetic acid (PA) and ethanol (EtOH) (Previously sanitized with irradiation)	Up to 240min	NR	.	3 log for PPV 10 min, >4 log for PPV 240 min
Vastel, 2007	Bone (femoral head)	14 donors	NR	NR	NR	Gamma irradiation (10 kGy)	NR	10 and 25 kGy irradiation had no impact compared to untreated, on bone elasticity	NR	NR

First Author, Year	Tissue Recovered	Number of samples	Cleaning of Tissue	Proportion of contamina-tion following recovery	Isolated Micro-organisms	Sanitization Method	Sanitization Parameters	Tissue Integrity	Proportion of positive cultures or microbial load	Logarithmic bioburden reduction
						Gamma irradiation (25 kGy)		or mechanical strength<br><br>Irradiation had moderate impact on some of the treated (defatted) bone specimens' elasticity and mechanical strength		
Hilmy, 2007	Femoral head and ring, tibia ring, humerus, patella tendon, tendon, fascia lata, ribs, struct, amnion	20 samples	Samples were shaken in 40 ml sterile saline for 10 min	Range of 2 to 22 cfu per sample	Gram positive, aerobic bacteria (no further details provided)	Irradiation at 2 kGy/h after sample inoculated with *Bacillus pumilus* spores (25 kGy)	NR	NR	Bone: 0% (0/10) positive & Amnion: 10% positive	Bone:0.82 average reduction (no colonies but CFU too low to give power to calculations)
Jones, 2007	Bone-patellar tendon-bone allografts		20 samples received 'BioCleanse™' treatment & 20 samples	NR	NR	'BioCleanse™ " treatment	NR	Mechanical properties (stiffness, cyclic creep, maximum	NR	NR

First Author, Year	Tissue Recovered	Number of samples	Cleaning of Tissue	Proportion of contamina-tion following recovery	Isolated Micro-organisms	Sanitization Method	Sanitization Parameters	Tissue Integrity	Proportion of positive cultures or microbial load	Logarithmic bioburden reduction
			untreated					force, ultimate stress) were not impacted by 'BioCleanse™' treatment.		
Schimizzi, 2007	Bilateral tibialis anterior tendons	17 donors	NR	NR	NR	Irradiation (20-26 kGy),	NR	Irradiation or 'BioCleanse' had no impact on allograft creep, failure load or failure stress. Irradiation and 'BioCleanse' increased allograft stiffness in the first cycle of testing.	NR	NR
						'BioCleanse'	4 h			
Bienek, 2007	Bone-Femoral shafts	NR	Originally, soft tissue, marrow and calcaneus bone removed. Then rinsed in	Originally NR	Bacteriophage ΦX174 or *Bacillus pumilus* spores	100% Ethylene oxide gas	0 minutes	NR	NR	0
							10 minutes			1.7
							30 minutes			>5.3
							240 minutes			>5.3

First Author, Year	Tissue Recovered	Number of samples	Cleaning of Tissue	Proportion of contamina-tion following recovery	Isolated Micro-organisms	Sanitization Method	Sanitization Parameters	Tissue Integrity	Proportion of positive cultures or microbial load	Logarithmic bioburden reduction
			distilled water repeatedly with sonication and heating (+58°C). Then frozen, lyophilized and packaged.			Gamma irradaiation (25 to 40 kGy)	NR			3.8 (log10 reduction in bacteriophage), >4.0 log10 reduction in *B. pumilus* spores
Grieb, 2006	Semitendinosus tendons	28 tendons	NR	100% (experimentally inoculated)	Inoculated with and tested for *Clostridium sordellii*, porcine parvovirus or Sindbis virus	Irradiation (18 kGy at -50°C)	NR	Decreased maximum force, increased ultimate strain, Young's modulus, and strain energy density<br><br>50 kGy irradition at -50°C with radioprotectant did not impact tendon strain, stress, Young's modulus, strain energy density,		Sindbis virus > 4.5; Porcine Parovirus 4.9,<br><br>Clostridium sordellii > 8.2
						Irradiation (50 kGy)				

First Author, Year	Tissue Recovered	Number of samples	Cleaning of Tissue	Proportion of contamina-tion following recovery	Isolated Micro-organisms	Sanitization Method	Sanitization Parameters	Tissue Integrity	Proportion of positive cultures or microbial load	Logarithmic bioburden reduction
								however, maximum force was reduced		
Mroz, 2006	Bone- Cylindrical cortical pins from femur and tibia	7 donors	NR	NR	NR	"BioCleanse™ "- hydrogen peroxide, isopropanol and other chemicals	2x protocol contact time to represent worst case scenario	"BioCleanse ™" decreased bone density but did not impact bone strength (axial compression, diametral compression, shear, three-point bend)	NR	NR
Akkus et al., 2005 (1)	Bone- Femurs	3 donors	NR	NR	NR	Irradiation 36.4 kGy	NR	Deformation energy reduced by 87% and elastic energy reduced by 26%	NR	NR

First Author, Year	Tissue Recovered	Number of samples	Cleaning of Tissue	Proportion of contamina-tion following recovery	Isolated Micro-organisms	Sanitization Method	Sanitization Parameters	Tissue Integrity	Proportion of positive cultures or microbial load	Logarithmic bioburden reduction
						1.5 M thiourea solution supplemented with calcium [IS] and protease inhibitors	4°C for 14 days	No change		
						Irradiation 36.4 kGy + 1.5 M thiourea solution supplemented with calcium [IS] and protease inhibitors	4°C for 14 days	Significant reduction in fracture energy and post-yield compared to no treatment. Significant improvement in fracture energy and post-yield compared to radiation treatment alone		
Akkus, 2005 (2)	Bone-Femoral shafts	3 donors	NR	NR	NR	Irradiation 36.4 kGy	NR	Negative impacts of irradiation are much greater on mechanical	NR	NR

First Author, Year	Tissue Recovered	Number of samples	Cleaning of Tissue	Proportion of contamina-tion following recovery	Isolated Micro-organisms	Sanitization Method	Sanitization Parameters	Tissue Integrity	Proportion of positive cultures or microbial load	Logarithmic bioburden reduction
								fatigue tests compared to monotonic loading.		
Scheffler, 2005	Bone-patellar tendon-bone	16 samples 8 donors	Soft tissues were dissected from allograft, and allografts were delipidated (chloroform-methanol) just prior to sterilization.	NR	NR	2% peracetic acid, 96% ethanol and aqua ad iniectabilia (ratio v/v/v 2/1/1).	4 h, under constant agitation and low pressure (200 mbar)	Peracetic acid sterilization did not impact mechanical properties (strain, creep, stiffness, failure load and max. elongation) of allografts.	NR	NR
Grieb, 2005	Bone-Cancellous bone dowels	NR	NR	100% (experimentally inoculated)	Inoculated with and tested for porcine parvovirus, Sindbis virus, *E. coli, S. aureus, B. subtilis, B. pumilus, A. niger, C. albicans, C. sordellii.*	Irradiation (18 kGy)	NR	NR	NR	NR
						Irradiation (50 kGy)		No significant change to bone integrity		

First Author, Year	Tissue Recovered	Number of samples	Cleaning of Tissue	Proportion of contamina-tion following recovery	Isolated Micro-organisms	Sanitization Method	Sanitization Parameters	Tissue Integrity	Proportion of positive cultures or microbial load	Logarithmic bioburden reduction
Baker, 2005	NR	400 samples	NR	NR	NR	Irradiation (1.03 kGy)	NR	NR	0% (0/300)	NR
						Irradiation (2.03 kGy)			0.33% (1/300)	
						Irradiation (3.09 kGy)			0% (0/300)	
						Irradiation (4.2 kGy)			0.33% (1/300)	
						Irradiaiton (5.4 kGy)			0% (0/300)	
Mitchell, 2004	Bone- Femurs		Stripped of soft tissue	NR	NR	22 specimens irradiated with 31.7 kGy	NR	Irradiated cortical bone specimens were less resistant to fatigue crack growth than non-irradiated controls.	NR	NR
Lomas, 2004	Entire knee joints	8 samples from 4 donors	Excess fat and connective tissues were removed	NR	NR	70% EtOH incubation for 24 h (4-10°C), repeated rinses with sterile PBS, then half of the samples were treated with 0.1% peracetic acid (pH 7-7.4, RT,	24 h EtOH, 3 h peracetic acid	Peracetic acid treatment did not render tendon cytotoxic or pro-inflammatory to human synovial fibroblasts in vitro.	NR	NR

First Author, Year	Tissue Recovered	Number of samples	Cleaning of Tissue	Proportion of contamination following recovery	Isolated Micro-organisms	Sanitization Method	Sanitization Parameters	Tissue Integrity	Proportion of positive cultures or microbial load	Logarithmic bioburden reduction
						3 h, with agitation). Rinsed with PBS.		Peracetic acid treatment did not effect tendon mechanical properties such as ultimate stress or Youngs modulus. However, ultimate strain was increased 41% with peracetic acid trreatment.<br><br>Although interfibre space was increased with peracetic acid treatment from 1.5% (control) to 24%, lyophilized ethylene oxide and irradiation		

First Author, Year	Tissue Recovered	Number of samples	Cleaning of Tissue	Proportion of contamina-tion following recovery	Isolated Micro-organisms	Sanitization Method	Sanitization Parameters	Tissue Integrity	Proportion of positive cultures or microbial load	Logarithmic bioburden reduction
								sterilization methods increased interfibre space to 28% and 79%, respectively.		
Moore, 2004	Bone powder, femoral cortex, iliac wedge, cancellous cubes, patellar bone-tendon-bone ligament	NR	Blood, cells, debris removed with high pressure deionized sterile water. Followed by lipid extraction and emulsification with hydrogen peroxide or ethanol.	100% (all inoculated with different viruses)	HIV-1, Bovine viral diarrhea, Reovirus type 3, Duck hepatitis B, Poliomyelitis, Canine Parvovirus	Ethylene oxide 214 mg/dL, 100% humidity, 30-33°C (no freeze-drying phase)	7 h	Complete inactivation of all viruses	0% positive	NR
Vastel, 2004	Bone-patellar tendon-bone Bone (femur) Bone (femoral heads; structure and morselized allografts)	18 femoral heads 14 donors	NR	NR	NR	Lipid extraction (acetone- not a sterilization method itself but often conducted along with other sterilization methods)	17 h	NR	NR	NR

First Author, Year	Tissue Recovered	Number of samples	Cleaning of Tissue	Proportion of contamina-tion following recovery	Isolated Micro-organisms	Sanitization Method	Sanitization Parameters	Tissue Integrity	Proportion of positive cultures or microbial load	Logarithmic bioburden reduction
						6 M Urea	3 h	Decreased speed of sound greatest for urea treatment. -Mechanical properties of bone: deformation at time of failure and stress to failure.		
						Irradiation 30 kGy	NR	Lipid extraction and irradiation did not impact measurements.		
Pruss,Kao et al 2003	Bone-Femoral heads	18	Cartilage removed	100% after inoculation	Human immunodeficiency virus type 2, Bovine viral diarrhoea virus, Pseudorabies virus, Hepatitis A virus,	Submerged in Ringer's Lactate solution and Heat at 82.5°C	≥ 15 min	Heat treatment inactivated all viruses to levels below the detection limit	0% positive (below threshold of virus detection)	BPV >4.26 HIV-2 >5.51 PRV >7.01 PV-1 >6.51 BVDV >4.51 HAV >6.51

First Author, Year	Tissue Recovered	Number of samples	Cleaning of Tissue	Proportion of contamina-tion following recovery	Isolated Micro-organisms	Sanitization Method	Sanitization Parameters	Tissue Integrity	Proportion of positive cultures or microbial load	Logarithmic bioburden reduction
					Poliomyelitis virus type 1, Bovine parvovirus					
Dunsmuir, 2003	Bone-Femoral heads	NR	NR	0% upon procurement<br><br>100% after inoculation	*Staphylococcus aureus*, *Bacillus subtilis* var. *globigii*	800 W microwave irradiation	0 minutes	NR	100% positive	NR
							1 min		100% positive	
							2 min		0% positive	
							3 min		0% positive	
							4 min		0% positive	
							5 min		0% positive	
							6 min		0% positive	

First Author, Year	Tissue Recovered	Number of samples	Cleaning of Tissue	Proportion of contamination following recovery	Isolated Micro-organisms	Sanitization Method	Sanitization Parameters	Tissue Integrity	Proportion of positive cultures or microbial load	Logarithmic bioburden reduction
Pruss, Gobel et al 2003	Spongiosa tissue from vertebral column, and epiphyses of femurs or tibiae	NR	Fat and connective tissue were manually removed. After specimens were cut, pieces were rinsed under high pressure (sterile water, 30 min, 37°C) to remove blood. Then, fat was removed with chloroform/methanol for 2 h with agitation.	100% after inoculation	Viruses- Human immunodeficiency virus type 2, Pseudorabies virus, Bovine virus diarrhoea virus, Hepatitis A virus, Poliomyelitis virus type 1, Porcine parvovirus	Peracetic acid-ethanol	5 - 240 min	NR	PRV 0%<br>PV1 0%<br>PPV 0%<br>HAV 0%<br>BVDV 0%<br>HIV-2 NR	PRV >4.19<br>PV-1 >5.57<br>PPV >4.96<br>HAV 2.87<br>BVDV >4.11<br>HIV-2 NR
					Hepatitis A virus	Peracetic acid-ethanol	5 - 240 min		100% positive for Hepatitis A virus	
					Staphylococcus aureus, Enterococcus faecium, Pseudomonas	Peracetic acid-ethanol	2 hours		0% positive bacterial or fungal cultures	>5 log reduction for all species except *Aspergillus*

First Author, Year	Tissue Recovered	Number of samples	Cleaning of Tissue	Proportion of contamina-tion following recovery	Isolated Micro-organisms	Sanitization Method	Sanitization Parameters	Tissue Integrity	Proportion of positive cultures or microbial load	Logarithmic bioburden reduction
					aeruginosa, Bacillus subtilis, Clostridium sporogenes, Mycobacterium terrae, Candida albicans, Aspergillus niger, Bacillus subtilis spores					>4 log reduction for *Aspergillus*, due to low starting inoculum
Tenholder, 2003	Bone - femur	14 femurs from 7 donors	Mechanical debridement, Negative pressure washing	NR	NR	Negative pressure washing and antibiotic irrigation	N/A	There was a small, inconsistent effect of negative pressure washing on compression tests, and no effect on other mechanical properties of bone.	N/A	NR
Dufrane, 2002	Fascia lata	NR	Washed with sterile	NR	NR	Defatted with acetone and	Listed in sanitization	Treatment did not render	NR	NR

First Author, Year	Tissue Recovered	Number of samples	Cleaning of Tissue	Proportion of contamina-tion following recovery	Isolated Micro-organisms	Sanitization Method	Sanitization Parameters	Tissue Integrity	Proportion of positive cultures or microbial load	Logarithmic bioburden reduction
			physiological saline (pulse lavage at RT). Later stripped of loose connective tissue (adipose, vessels, nerves).			ethanol, then prion inactivation with 1 N NaOH (1 h). Treated with NaCl (1 h) , hydrogen peroxide (15 h), and freeze-dried (3 days). Allografts were then packaged and irradiated (25 kGy).	method column.	allografts cytotoxic to human fibroblasts. In addition, growth and spreading of fibroblasts loaded onto allografts was observed after 3 and 7 days.		
Pruss, Kao *et al* 2002	Cancellous and cortical bones	NR	Removal of muscle and connective tissue	100% after inoculation	Human immunode-ficiency virus type 2	Irradiation 34 kGy at -30 ± 5°C	NR	NR	NR	In bone log reduction >4.0
					Bovine viral diarrhoea virus					In bone log reduction >5
					Pseudorabies virus					In bone log reduction >4.4
					Hepatitis A virus					In bone log reduction >7.2

First Author, Year	Tissue Recovered	Number of samples	Cleaning of Tissue	Proportion of contamina-tion following recovery	Isolated Micro-organisms	Sanitization Method	Sanitization Parameters	Tissue Integrity	Proportion of positive cultures or microbial load	Logarithmic bioburden reduction
					Polio virus type 1					In bone log reduction >5.9
					Bovine parvovirus					In bone log reduction >3.7
Pruss, 2001	Spongiosa tissue from columna vertebralis, and epiphyses of femur, humerus or tibia	NR	Fat and connective tissue were removed. After specimens were cut, pieces were rinsed (sterile water, 30 min, 37°C) to remove blood. Then, fat was removed with chloroform/methanol for 2 h with agitation, flushed 8x with methanol, then ultrasonic water bath (15 min). Samples were not freeze-dried.	0% positive on newly cut and cleaned bone cuboids<br><br>100% after inoculation	Inoculation with: *Staphylococcus aureus*, *Enterococcus faecium*, *Pseudomonas aeruginosa*, *Bacillus subtilis*, *Bacillus subtilis* spores, *Clostridium sporogenes*, *Mycobacterium terrae*, *Candida albicans* and *Aspergillus*	Peracetic acid-ethanol (2:1:1 of peracetic acid:ethanol: physiological salt solution)	2 or 4 h	Bacteria and fungi were completely inactivated by 2 h of peracetic acid-ethanol treatment.	0% positive	*Staphylococcus* >8.23<br>*Enterococcus* >6.89<br>*Pseudomonas* >7.89<br>*Bacillus* >6.46<br>*Bacillus* spores >5.81<br>*Clostridium* >7.63<br>*Mycobacteria* >6.47<br>*Candida* >7.27<br>*Aspergillus* >4.71

First Author, Year	Tissue Recovered	Number of samples	Cleaning of Tissue	Proportion of contamina-tion following recovery	Isolated Micro-organisms	Sanitization Method	Sanitization Parameters	Tissue Integrity	Proportion of positive cultures or microbial load	Logarithmic bioburden reduction
					niger spores.					
Akkus, 2001	Bone- Cortical bone from femora	40 samples	NR	NR	NR	Irradiation (27.5 kGy) on dry ice	NR	Irradiation decreased fracture toughness, work-to-fracture, accoustic emissions (measure of elastic properties) and ability to sustain damage.	NR	NR
Lomas, 2001	Bilateral tibialis anterior tendons<br>Bone- Femoral shafts<br>Semitendinosus tendons<br>Bone- Cylindrical cortical pins from femur and tibia	NR	0.9% saline, 56-60°C, 3 h, and then soft tissues dissected away, and samples refrozen at -80°C.<br><br>Bone samples then cut and pulverized (frozen), the powder was	NR	NR	Ethylene oxide (37°C, standard cycle with aeration)	NR	Small particle TNF-A and IL-6 induction	NR	NR
						Ethylene oxide (37°C, standard cycle with no aeration)	NR	No cytokine induction		
						Gamma irradiation (25-33 kGy)	NR	No cytokine induction		

First Author, Year	Tissue Recovered	Number of samples	Cleaning of Tissue	Proportion of contamina-tion following recovery	Isolated Micro-organisms	Sanitization Method	Sanitization Parameters	Tissue Integrity	Proportion of positive cultures or microbial load	Logarithmic bioburden reduction
			demineralized (0.6 N HCl, 4-10°C) and then delipidated (chloroform/methanol, 1 h), rinsed with methanol and then water. The powder was frozen and lyophylized.			Autoclaving	134°C, 20 min	No cytokine induction		
Clavert, 2001	Tendons of the long head of the biceps brachii.	16 samples	NR	NR	NR	NR	NR	Relaxation test, uniaxial freezing test. One freeze-thaw cycle did not impact tendon relaxation. However, ultimate tensile failure and Young's modulus were impacted.	NR	NR
Hernigou, 2000	Bone- Tibiae, femurs	NR	Intramedullary content removed	100%	HIV-1/LAI	Heat at 50°C	30 minutes	49 Wmax (Wmax = max load that can		1.12 log reduction

First Author, Year	Tissue Recovered	Number of samples	Cleaning of Tissue	Proportion of contamina-tion following recovery	Isolated Micro-organisms	Sanitization Method	Sanitization Parameters	Tissue Integrity	Proportion of positive cultures or microbial load	Logarithmic bioburden reduction
								be applied)		
						30 kGy at -80°C	NR	46 Wmax		4.2 log reduction
						Heat at 50°C (30 min) then 30 kGy at 80°C	NR	43 Wmax		5.19 log reduction
						30 kGy at –80 °C, followed by 50°C	NR	47 Wmax		7.16 log reduction
						50 kGy for 30 min and 30 kGy	NR	29 Wmax		8.43 log reduction
						50 kGy at -80°C	NR	17 Wmax		7.02 log reduction
Moreau, 2000	Bone-Femoral heads	NR	NR. After core samples were drilled, the cartilaginous articular surface was removed. Subset of samples were incubated in 5% NaHCO3 at	NR	NR	Irradiation 25 kGy	NR	Irradiation induced a 2 to 3-fold increase in lipid peroxidation.<br><br>Raw bone slices (with lipids) did not induce cell	NR	NR

First Author, Year	Tissue Recovered	Number of samples	Cleaning of Tissue	Proportion of contamina-tion following recovery	Isolated Micro-organisms	Sanitization Method	Sanitization Parameters	Tissue Integrity	Proportion of positive cultures or microbial load	Logarithmic bioburden reduction
			50°C for 24 h, and then defatted in acetone (8 h), and 40% ethanol (4 h).					death of osteoblast-like cells (Soas-2), however, irradiated slices (with lipid) induced a large increase in cell death. Defatted and irradiated slices did not induce cell death.		

Table 8: Recovery and Processing of Allografts for Clinical Studies

First Author, Year	Type of Recovered Tissue	Number of Samples	Cleaning of Tissue	Bioburden following recovery	Isolated Micro-organisms	Sanitization Method	Sanitization Period	Tissue Integrity	Proportion of positive cultures or microbial load	Logarithmic bioburden reduction
Sun, 2012	Hamstring tendon	78 samples	NR	NR	NR	Irradiation (2.5 Mrad or 25 kGy), No irradiation	NR	See Table 9	NR	NR
Sun, 2009	Bone-patellar tendon-bone	158 samples	NR	NR	NR	Irradiated (2.5 Mrad or 25 kGy) allograft, non-irradiated allograft, non-irradiated autograft	NR	See Table 9	NR	NR
Indelicato, 2013	Bone-patellar tendon-bone	67 samples	NR	NR	NR	Sterilized allografts (BioCleanse-sterilized)<br>Aseptic allografts (BTB allografts)	NR	NR	NR	NR
Kim, 2011	Bone (iliac bone) and soft tissue (fascia lata)	31 samples	NR	NR	NR	Gamma irradiation (25 KGy)	NR	NR	NR	NR
Gajiwala, 2003	Bone- iliac crest, femoral head or long bone segments	41 samples	Washed with water and pasteurized (60°C, 3 h), soft tissue removed, defatted, freeze-dried, demineralized	NR	NR	25 kGy radiation	NR	NR	NR	NR
Krasny, 2013	Corticospongious bone	68 samples	Alcohol solution for	NR	NR	Irradiation (35 kGy) for	NR	NR	NR	NR

First Author, Year	Type of Recovered Tissue	Number of Samples	Cleaning of Tissue	Bioburden following recovery	Isolated Micro-organisms	Sanitization Method	Sanitization Period	Tissue Integrity	Proportion of positive cultures or microbial load	Logarithmic bioburden reduction
	blocks from the iliac ala, demineralized bone matrix from the compact bone of diaphysis		spongious bone; 0.6M HCL for demineralized bone matrix			corticospongious bone;				
						Irradiation (25 kGy) for demineralized bone matrix				
Guo, 2012	Bone-patellar tendon-bone	187 samples	NR .	NR	NR	None- autografts	NR	See Transplantation outcomes	NR	NR
						None- Fresh Frozen allografts	NR			
						Gamma irradiation (no dose was recorded)	20 hours			
Mehendale, 2009	Bone (femoral heads)	49 samples	NR	NR	NR	Irradiation (25 or 50 kGy)	NR	See Transplantation outcomes	NR	NR
Galia, 2009	Bone-morselized	41 samples	Freeze-dried, defatted	NR	NR	"Sterilized"	NR	NR	NR	NR
Khoo, 2006	Bones-femoral shafts,	45 samples	NR	NR for original allograft	NR	Irradiation (>25 kGy)	NR	NR	NR - Only tested after transplantat	NR

First Author, Year	Type of Recovered Tissue	Number of Samples	Cleaning of Tissue	Bioburden following recovery	Isolated Micro-organisms	Sanitization Method	Sanitization Period	Tissue Integrity	Proportion of positive cultures or microbial load	Logarithmic bioburden reduction
	proximal humerus, femur, tibia, distal femur								ion, no testing following irradiation.	
Pruss Perka, 2002	Cancellous bone, cortical bone, amnion, demineralized bone matrix, ligament/ tendons	2060 samples	Cleaned, defatted (cancellous)	NR	NR	Peracetic acid-ethanol	NR	NR	NR	NR
