# Supplementary material for: Disinfection of human musculoskeletal allografts in tissue banking: a systematic review
Source: Cell Tissue Bank. 2016 Sep 24;17(4):573–84. doi: 10.1007/s10561-016-9584-3 (PMC5116033; doi:10.1007/s10561-016-9584-3)
Supplement: Supplementary file 7 — Supplementary material 7 (PDF 92 kb) [file 10561_2016_9584_MOESM7_ESM.pdf]

Appendix G:

Table 9: Transplantation Outcomes for Clinical Studies

First Author, Year	Tissue Recovered	Decontamination method	Surgery Type	Number of Patients	Number of Samples Transplanted	Patient Morbidity/ Mortality	Adverse events[1]	Proportion of successful allograft transplantation	Clinical Outcome Metric
Sun, 2012	Hamstring tendon Bone-patellar tendon-bone	Non-irradiated allograft,	Anterior cruciate ligament reconstruction	76 but 69 were available for full evaluation	38 non-irradiated allografts	NR	None	69/76 (patients were lost to follow-up due to migration, personal reasons, lack of interest, bad clinical outcome of ACL reconstruction etc.)	Significant differences: Lachman test, ADT, pivot shift test, instrumented KT-2000 arthrometer testing.<br><br>No difference: vertical jump test, one-leg hop test, ROM of knee, Cincinnati knee score, IKDC Subjective Knee Form, Tegner activity score, Modified Lysholm knee scoring scale, the standard knee ligament evaluation form of the IKDC.
		Irradiated (2.5 Mrad or 25 kGy) allograft			31 irradiated allografts	NR	None		
Sun, 2009	Bone-patellar tendon-bone	Irradiated (2.5 Mrad or 25 kGy) allograft, non-irradiated	Anterior cruciate ligament reconstruction	99	99	NR	0 early post-operative infection or	100% (98/98)	Statistically significant differences between comparing

First Author, Year	Tissue Recovered	Decontamination method	Surgery Type	Number of Patients	Number of Samples Transplanted	Patient Morbidity/ Mortality	Adverse events[1]	Proportion of successful allograft transplantation	Clinical Outcome Metric
		allograft, non-irradiated autograft					wound problems; 1 patient (2.9%) in the Non-Irradiated-Allo group had a late infection that needed antibiotic treatment		irradiated allograft to non-irradiated allograft or autograft at 31 mos follow-up: the Lachman test, anterior drawer test, pivot shift test, KT-2000 arthrometer testing<br><br>No difference among the 3 groups: Harner's vertical jump and Daniel's one-leg hop tests, ROM of knee, Tegner activity score and Lysholm knee scoring scale, Cincinnati knee score, the standard knee ligament evaluation form of the International Knee Documentation Committee (IKDC)

First Author, Year	Tissue Recovered	Decontamination method	Surgery Type	Number of Patients	Number of Samples Transplanted	Patient Morbidity/ Mortality	Adverse events[1]	Proportion of successful allograft transplantation	Clinical Outcome Metric
Indelicato, 2013	Bone (iliac bone) and soft tissue (fascia lata)	Aseptic allografts (BTB allografts)	Anterior cruciate ligament reconstruction	24	24 aseptic allografts	None	None	100% successful transplants	No difference between groups (ROM, IKDC, KT-1000 kneww arthrometer)
		Sterilized BTB allografts (BioCleanse-sterilized)		43	43 BioCleanse-sterlized allografts	None	None	100% successful transplants	
Kim, 2011	Bone- iliac crest, femoral head or long bone segments	Gamma irradiation (25 KGy)	Endonasal transsphenoidal surgery and sellar reconstruction using allografts following tumor removal.	31	31	NR; although 3/31 had post-op CSF leak (two required a second intervention) and postoperative meningitis in one patient (3.2%)	NR	31/31 (100%)	Overall, postoperative cerebrospinal fluid (CSF) leaks occurred in three patients (9.7%) and postoperative meningitis in one patient (3.2%). Postoperative meningitis was improved with the use of antibiotics and prolonged CSF diversion.
Gajiwala, 2003	Hamstring tendon	25 kGy radiation	Tumor surgery (morselized allograft for reconstruction)	41	32 utilized morselized bone allografts and were discussed	9.8% (4/41 cases of infections)	Deep infection	90.2% (37/41)	24/25 patients showed incorporation of allograft by 6 to 9 months

First Author, Year	Tissue Recovered	Decontamination method	Surgery Type	Number of Patients	Number of Samples Transplanted	Patient Morbidity/ Mortality	Adverse events[1]	Proportion of successful allograft transplantation	Clinical Outcome Metric
Krasny, 2013	Corticospongious bone blocks from the iliac ala, demineralized bone matrix from the compact bone of diaphysis	Irradiation (35 kGy) for corticospongious bone;	Open sinus floor elevation, post-extraction alveoli augmentation and reconstruction of atrophied alveolar process.	68	NR	None	None	100%	NR
		Irradiation (25 kGy) for demineralized bone matrix							
Guo, 2012	Bone-patellar tendon-bone	None- autografts	Anterior cruciate ligament reconstruction	187 at the beginning; 142 successfully followed-up	41 had autografts	NR		100% (41/41) autografts	More anterior laxity in the gamma-irradiated allograft group compared with the fresh frozen allograft and the autograft<br>No difference among the 3 groups
		None- Fresh Frozen allografts			33 had fresh-frozen allografts	NR	3/33 cases of acute synovitis after surgery in the fresh-frozen allograft group within 1 week postoperatively	30/33 fresh-frozen allografts	

First Author, Year	Tissue Recovered	Decontamination method	Surgery Type	Number of Patients	Number of Samples Transplanted	Patient Morbidity/ Mortality	Adverse events[1]	Proportion of successful allograft transplantation	Clinical Outcome Metric
		Gamma irradiation (no dose was recorded)			68 gamma-irradiated allografts	NR	NR	62/68 gamma-irradiated allografts	
Mehendale , 2009	Bone (femoral heads)	irradiation (25 or 50 kGy)	Revision hip replacements	49	50	NR	Infection in 1 case	38/50 (76%)	Clinically a good or excellent outcome with absence of pain was achieved in 35 patients (70%); All the 6 patients in which the acetabular component was loose had moderate to severe pain.
Galia, 2009	Bone-morselized	NR	Revision total hip arthroplasty	42	42	2.4% (1/42) superficial skin infection 6 months post-surgery	NR	100% (42/42)	Allograft was remodelled and incorporated (90%) and also produced (90%) adequate outcome
Khoo, 2006	Bone- iliac crest, femoral head or long bone segments	Irradiation (>25 kGy)	Femurs, tibiae, humerus	42	45 allografts in 42 patients. However, 26 patients were followed-up for ≥ 2 years.	No mortalities related to allograft im-plantation.	Infection in 2 of 34 allograft implants. One remedied with	94.1% (32/34)	No early deep infections, two late infections

First Author, Year	Tissue Recovered	Decontamination method	Surgery Type	Number of Patients	Number of Samples Transplanted	Patient Morbidity/ Mortality	Adverse events[1]	Proportion of successful allograft transplantation	Clinical Outcome Metric
							antibiotics, the other (hinged knee prosthesis-allograft) required above-knee amputation		
Pruss Perka, 2002	Bone	Peracetic acid-ethanol	"Transplants" (many)	2,592 were studied	16,823, however, 3,087 were included in study	0% (0/1,205) infection transmissio n, 0.75% (9/1,205) secondary infections or wound healing defects	No adverse events were observed.	99.25% (1,196/1205)	Allogeneic tissue transplants treated by PES show a good up to very good clinical efficacy. No clinically relevant side effects.

[1]Only including adverse events as a result of microbial contamination attributed to tissue allograft

* denotes statistically significant results
